# Animal-Assisted Therapy in Dementia: A Systematic Review and Meta-Analysis of Cognitive, Affective, and Behavioral Outcomes

**DOI:** 10.3390/jcm15176654

**Published:** 2026-08-28

**Authors:** Man Amanat, Mona Salehi, Mahdieh Saeidi, Larissa Villacres Mosquera, Mohsan Ali, Jai Ahuja, Nicole Theis-Mahon, Omar Fattal, Sasidhar Gunturu

**Affiliations:** 1 Department of Neurology, Mayo Clinic, Rochester, MN 55902, USA; 2 Department of Psychiatry, University of Minnesota, Minneapolis, MN 55455, USA; 3 Department of Psychiatry, BronxCare Health System, New York, NY 10457, USA; 4 Department of Neuropsychiatry, Prisma Health, Columbia, SC 29212, USA; 5 NYC Health and Hospitals, New York, NY 10004, USA; 6 Department of Psychiatry, Icahn School of Medicine at Mount Sinai, New York 10028, NY, USA

**Keywords:** dementia, animal therapy, BPSD, cognition, systematic review

## Abstract

**Background/Objectives:** Behavioral and psychological symptoms of dementia (BPSD) are common and represent major drivers of caregiver burden and healthcare utilization. Animal-assisted therapy (AAT) is used as a non-pharmacological intervention in dementia care, yet quantitative evidence of its efficacy remains inconsistent. We systematically evaluated the effects of AAT on cognitive, affective, and behavioral outcomes in individuals with dementia. **Methods:** Following PRISMA 2020 guidelines, we searched seven electronic databases from inception through 27 September 2025 for studies evaluating AAT in dementia. Randomized and non-randomized controlled studies were included in quantitative synthesis. Risk of bias was assessed with the Cochrane Risk of Bias 2 and ROBINS-I tools. Random-effects meta-analyses used standardized mean differences (SMDs), with sensitivity, influence, subgroup, and publication bias analyses conducted where appropriate. **Results:** Fifty-nine studies met inclusion criteria, including 24 controlled studies with 1881 participants. AAT was associated with reduced overall BPSD severity, although this effect was sensitive to study quality and was not retained after excluding high-risk studies. AAT significantly improved depressive symptoms (SMD, −0.79, 95%CI, −1.33 to −0.26) and cognitive performance (SMD, 1.17; 95%CI, 0.31 to 1.79), with substantial heterogeneity; both effects persisted after excluding studies at high or serious risk of bias. No significant effects were observed for agitation, quality of life, or activities of daily living. Benefits were observed primarily in live animal interventions; robotic subgroups comprised a few studies and were underpowered to detect an effect. **Conclusions:** AAT may confer meaningful benefits for mood and cognitive engagement in dementia, particularly with live animals, but confidence is limited by methodological heterogeneity and the predominance of studies at moderate to serious risk of bias.

## 1. Introduction

Dementia represents a rapidly expanding global health challenge, currently affecting more than 55 million individuals worldwide [1]. While cognitive decline is the clinical hallmark, the disease course is frequently dominated by behavioral and psychological symptoms of dementia (BPSD), including depression, agitation, apathy, and psychosis. These non-cognitive symptoms affect over 90% of individuals during the course of illness [2] and are major drivers of caregiver burden, early institutionalization, and increased healthcare utilization [3]. Pharmacological management, particularly with atypical antipsychotics, is limited by modest efficacy and safety concerns [4,5]. Consequently, contemporary clinical guidelines emphasize non-pharmacological interventions as a core component of BPSD management [6].

Among non-pharmacological approaches, animal-assisted therapy (AAT) has been increasingly implemented in dementia care settings. AAT is proposed to leverage the human–animal bond to provide multisensory stimulation and non-judgmental social engagement, potentially addressing sensory deprivation, loneliness, and emotional distress in individuals with dementia [7].

Despite its growing use, the quantitative evidence base for AAT remains equivocal. Prior systematic reviews have reported inconsistent findings across behavioral, emotional, and cognitive domains [7,8,9]. A key limitation of the existing literature is the lack of explicit differentiation between interventions involving live animals and those utilizing robotic animal surrogates, limiting conclusions regarding whether their effects are comparable or distinct. In addition, many primary studies are small and methodologically heterogeneous, and prior reviews have not systematically assessed risk of bias or conducted quality-informed sensitivity analyses, constraining interpretation of reported effects.

In this context, we conducted a systematic review and meta-analysis to evaluate the effects of AAT on cognitive, affective, and behavioral outcomes in individuals with dementia, incorporating both randomized and non-randomized controlled studies for quantitative synthesis.

## 2. Methods

This study was conducted in accordance with the Preferred Reporting Items for Systematic Reviews and Meta-Analyses (PRISMA) 2020 guidelines (Supplementary Table S1). The study protocol was developed a priori by the authors and registered in the PROSPERO database (registration number: CRD420251059300).

### 2.1. Search Strategy

A comprehensive systematic literature search was conducted to identify studies evaluating the effects of AAT on clinical outcomes in individuals with dementia. Searches were performed in MEDLINE, Embase, APA PsycINFO, CAB Abstracts, Scopus, Web of Science Core Collection, and Cochrane CENTRAL. All databases were searched from inception through 27 September 2025.

The search strategy combined controlled vocabulary terms (e.g., MeSH and Emtree) and free-text keywords related to dementia and animal-assisted interventions. Searches were limited to English-language publications. Full search strategies for each database are provided in Supplementary Table S2.

### 2.2. Eligibility Criteria

The review question was specified a priori using the PICOS framework. Population: adults with dementia or major neurocognitive disorder, of any subtype and severity, as defined by the original study authors; Intervention: animal-assisted therapy or animal-facilitated intervention, delivered using either live animals or robotic animal companions; Comparator: treatment as usual, an attention- or time-matched active control, or no intervention; Outcomes: overall BPSD severity, depressive symptoms, cognitive performance, agitation, quality of life, and activities of daily living; Study Design: Randomized and non-randomized controlled studies were eligible for quantitative synthesis; non-controlled designs were eligible for descriptive synthesis only.

Dementia was diagnosed clinically in all included studies; no study used biomarker confirmation. Both controlled and non-controlled study designs were eligible for inclusion. Non-controlled studies were included for descriptive synthesis. Non-dementia populations, qualitative-only studies, non-human studies, conference abstracts, dissertations, and review articles were excluded. For overlapping samples, the most complete dataset was retained. Independent non-overlapping samples (e.g., multiple phases or facilities) were treated as separate comparisons.

Two reviewers (MoA and JA) independently screened titles and abstracts, followed by full-text assessment of potentially eligible studies. Disagreements were resolved through discussion and consensus, with consultation of a third reviewer when necessary (MaS).

### 2.3. Data Extraction

Two authors independently extracted data using a standardized data extraction form (LM and MoA). Extracted information included study characteristics (first author, year, country, setting, and design), participant characteristics (sample size, sex distribution, and dementia type and severity), intervention characteristics (animal type, duration, frequency, and comparator), outcome measures, and numerical outcome data required for meta-analysis (means, standard deviations, and sample sizes). Any discrepancies in data extraction were resolved by consensus after review of the original publications and consultation of a third reviewer (MoS) when necessary.

### 2.4. Risk of Bias Assessment

Risk of bias for randomized controlled trials was assessed using the revised Cochrane Risk of Bias tool (RoB 2), which evaluates bias arising from the randomization process, deviations from intended interventions, missing outcome data, outcome measurement, and selective reporting.

Non-randomized controlled studies were assessed using the Risk Of Bias In Non-randomized Studies of Interventions (ROBINS-I) tool, covering bias due to confounding, participant selection, classification of interventions, deviations from intended interventions, missing data, outcome measurement, and selective reporting.

Two reviewers (MaS and JA) independently performed all risk-of-bias assessments. Discrepancies were resolved by discussion and consultation of a third reviewer (MoS) when necessary. Overall judgments were categorized as low risk, some concerns/moderate risk, or high/serious risk of bias.

### 2.5. Certainty of Evidence

The certainty of evidence for each pooled outcome was assessed using the GRADE approach. Bodies of evidence derived from randomized trials began at high certainty and were rated down for risk of bias, inconsistency, indirectness, imprecision, and publication bias, with each domain rated as not serious, serious, or very serious. Certainty was categorized as high, moderate, low, or very low. Assessments were performed independently by two reviewers (MA and MoS), with disagreements resolved by discussion.

### 2.6. Outcomes

Six outcome domains were prespecified, including overall BPSD severity, depressive symptoms, cognitive performance, agitation, quality of life, and activities of daily living. Overall BPSD severity was defined as the total score of a multidomain neuropsychiatric rating instrument. Individual measurement instruments were not prespecified; any validated instrument reported by the primary studies as assessing a domain of interest was eligible, and data were pooled by domain rather than by instrument. The instruments contributing to each domain are reported in the corresponding Results subsections.

### 2.7. Statistical Analysis

Random-effects meta-analyses were performed for outcomes reported by at least two controlled studies using standardized mean differences (SMDs) with 95% confidence intervals (CIs). As outcome measures differed in scale orientation and coding, effect directions were interpreted as displayed in the forest plots. For depressive symptoms, total BPSD scores, agitation, and ADL, negative SMDs favored AAT, whereas for cognition and quality of life, positive SMDs favored AAT. Statistical heterogeneity was assessed using the Cochran Q test and quantified with the I^2^ statistic, with values above 50% indicating substantial heterogeneity.

Pre-specified subgroup analyses were conducted to explore sources of heterogeneity based on intervention type (live animal vs. robotic animal) and outcome measurement scale when applicable. Influence analyses were performed using leave-one-out methods to assess the robustness of pooled estimates. Sensitivity analyses were conducted by excluding studies judged to be at high or serious risk of bias. Additionally, we performed sensitivity analyses restricted to randomized controlled trials for each outcome to assess whether pooled estimates were robust to the inclusion of non-randomized evidence. For outcomes with at least 10 contributing studies, potential publication bias and small-study effects were evaluated using visual inspection of funnel plots with pseudo-95% confidence limits and Egger’s regression test.

For overall BPSD severity, only total scores from multidomain neuropsychiatric instruments were pooled; subscale scores were not summed or combined across instruments. Depressive symptoms and agitation were analyzed as separate outcome domains rather than as BPSD subscales. All statistical analyses were performed using Review Manager (RevMan) version 11.1.0 (The Cochrane Collaboration, 2026) and Stata version 19 (StataCorp LLC, College Station, TX, USA).

## 3. Results

### 3.1. Included Studies

**Figure 1:**
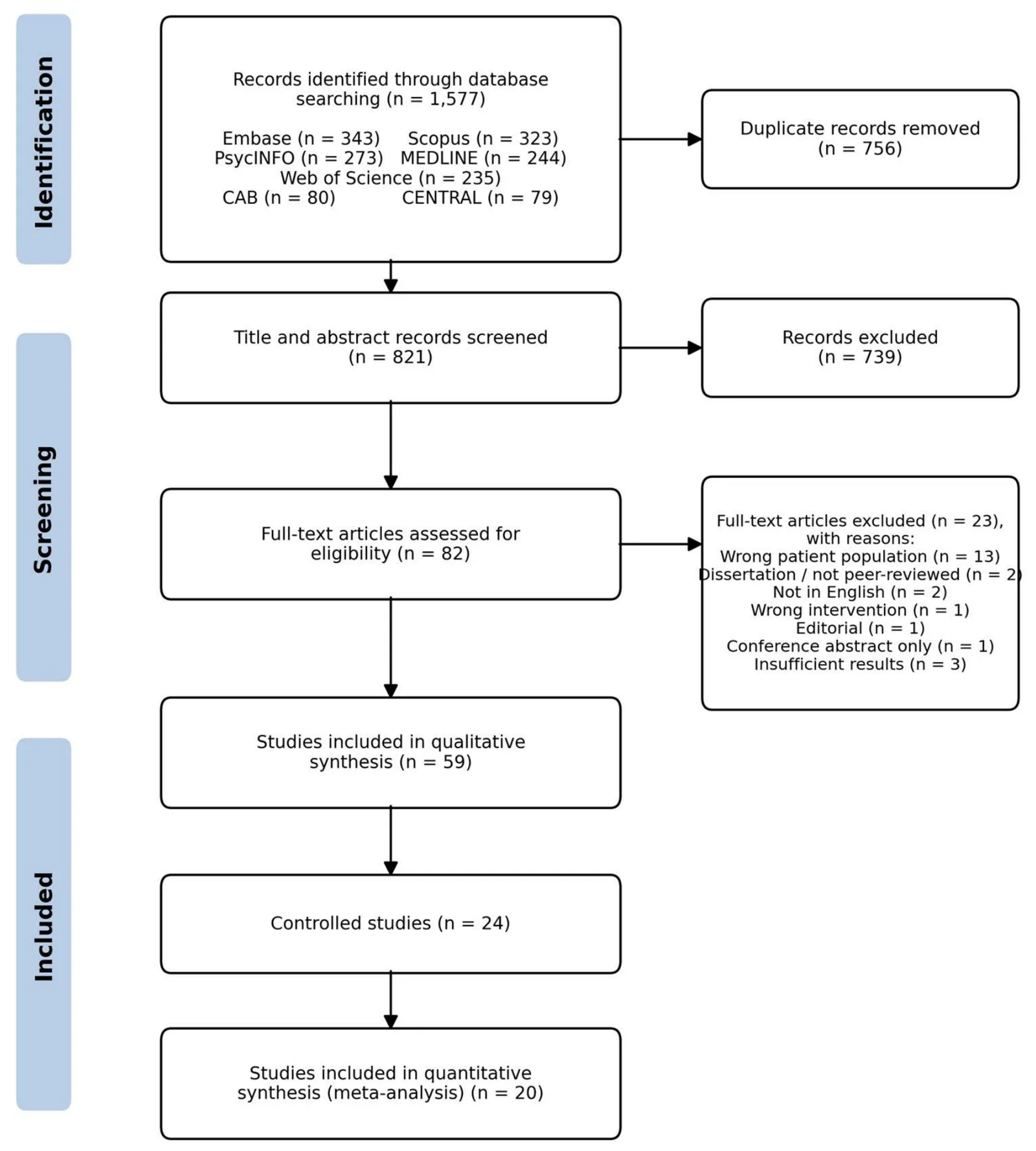
PRISMA 2020 flow diagram of study identification, screening, and inclusion.

The study selection process is summarized in the PRISMA flow diagram (Figure 1). Fifty-nine studies met eligibility criteria, including 35 non-controlled studies summarized in Supplementary Table S3.

### 3.2. Characteristics of Controlled Studies

Twenty-four controlled studies were included, one of which reported two independent phases with non-overlapping samples [10]. Across studies, 1881 participants were enrolled (Table 1). Seventeen studies were randomized [10,11,12,13,14,15,16,17,18,19,20,21,22,23,24,25,26] and seven were non-randomized [27,28,29,30,31,32,33]. Studies were conducted in 10 countries, namely Italy (*n* = 5) [12,21,27,30,31]; Spain (*n* = 4) [10,13,25,32]; United States (*n* = 4) [14,15,20,26]; Germany (*n* = 3) [16,29,33]; Norway (*n* = 2) [18,19]; Australia (*n* = 2) [17,24]; and one study each from South Korea [11], Japan [28], New Zealand [22], and The Netherlands [23].

Dementia was ascertained clinically in all 24 controlled studies. Diagnostic criteria were reported inconsistently. Diagnostic criteria were DSM-IV [16,20,28] and ICD-10 [27,31]. In the remaining studies, dementia was based on a diagnosis documented in the clinical record or on a cognitive screening threshold, most commonly the MMSE or a national equivalent, without stated criteria.

Dementia subtype was reported inconsistently. Alzheimer’s disease predominated where subtype was specified, with vascular dementia the next most frequent. Three studies enrolled mixed cohorts including non-Alzheimer’s, non-vascular subtypes: frontotemporal dementia, Parkinson’s disease dementia, and Korsakoff’s syndrome [23]; Lewy body dementia [17]; and secondary dementias [31]. No study reported outcomes stratified by dementia subtype.

Most studies evaluated live dog-assisted interventions (*n* = 19) [10,11,12,13,15,16,18,19,21,23,24,25,26,27,29,30,31,32,33]. Robotic pet interventions were tested in 5 studies [10,17,20,22,23]. Equine-assisted therapy [14] and mixed animal interventions (cats/dogs) [28] were each assessed in one study.

Alzheimer’s disease-only samples were clearly specified in 4 studies [10,12,21,30]. Across the remaining studies, dementia was mixed, a broader neurocognitive disorder, or not fully subtype-specified.

### 3.3. Risk of Bias

Among randomized trials, seven were judged to be at high risk of bias [11,13,14,18,19,20,23], while the remaining 10 were judged to have some concerns [10,12,15,16,17,21,22,24,25,26] (Supplementary Figure S1A). Inadequate reporting of randomization and allocation concealment were the most common limitations. Furthermore, bias was commonly identified in the domains of deviations from intended interventions and outcome measurement, largely due to the inherent difficulty of blinding participants and personnel in animal-assisted interventions (Supplementary Table S4).

The overall risk of bias among non-randomized trials was judged as moderate in four studies [27,29,30,31] and serious in three [28,32,33] (Supplementary Figure S1B). Serious ratings were primarily driven by concerns regarding confounding and selection into the intervention, such as allocation based on participant preference for animals [28]. Additionally, outcome measurement bias was a factor in designs where outcome assessors could not be blinded to the presence of a dog, such as the video-coded behavioral outcomes utilized in Wesenberg et al. [33] (Supplementary Table S4).

### 3.4. Meta-Analysis

Of the 24 controlled studies assessing clinical outcomes, 20 contributed data to meta-analyses across six domains: overall BPSD, depressive symptoms, cognition, agitation, quality of life, and activities of daily living. The remaining four assessed outcomes were outside these domains and were summarized narratively rather than included in the quantitative synthesis.

### 3.5. Overall BPSD

Five controlled publications contributed six independent comparisons evaluating total BPSD severity, comprising 341 participants in the AAT groups and 275 participants in control conditions. One publication [10] included two intervention phases, each with three independent study arms, which were treated as separate comparisons because participants did not overlap across phases. BPSD outcomes were assessed using the Neuropsychiatric Inventory (NPI; three comparisons), the Multidimensional Observation Scale for Elderly Subjects (MBPC; one comparison), the Neuropsychiatric Hospital Behavior Problem Scale (NHBPS; one comparison), and the Behavioral Pathology in Alzheimer’s Disease Rating Scale (Behave-AD; one comparison).

**Figure 2:**
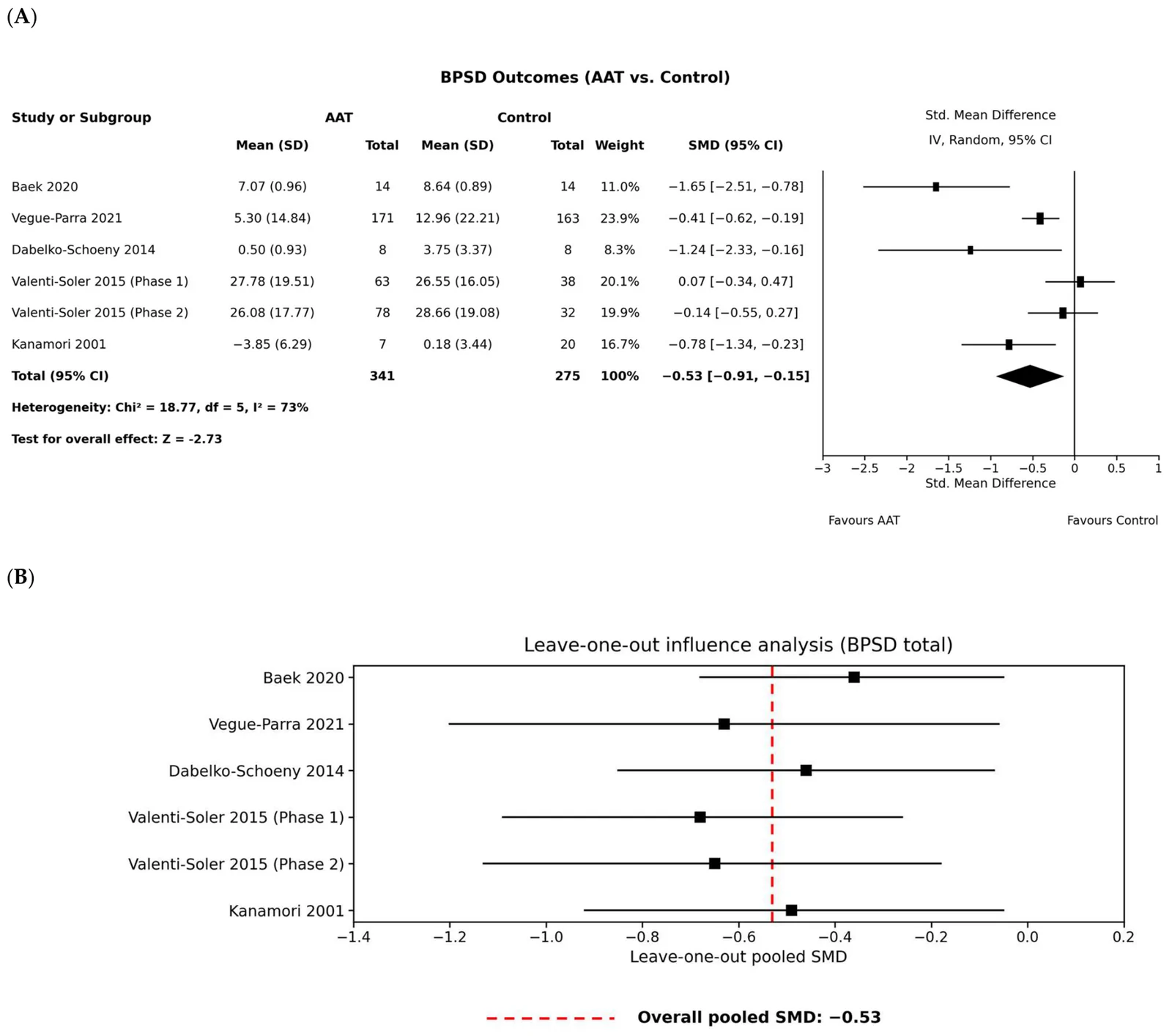

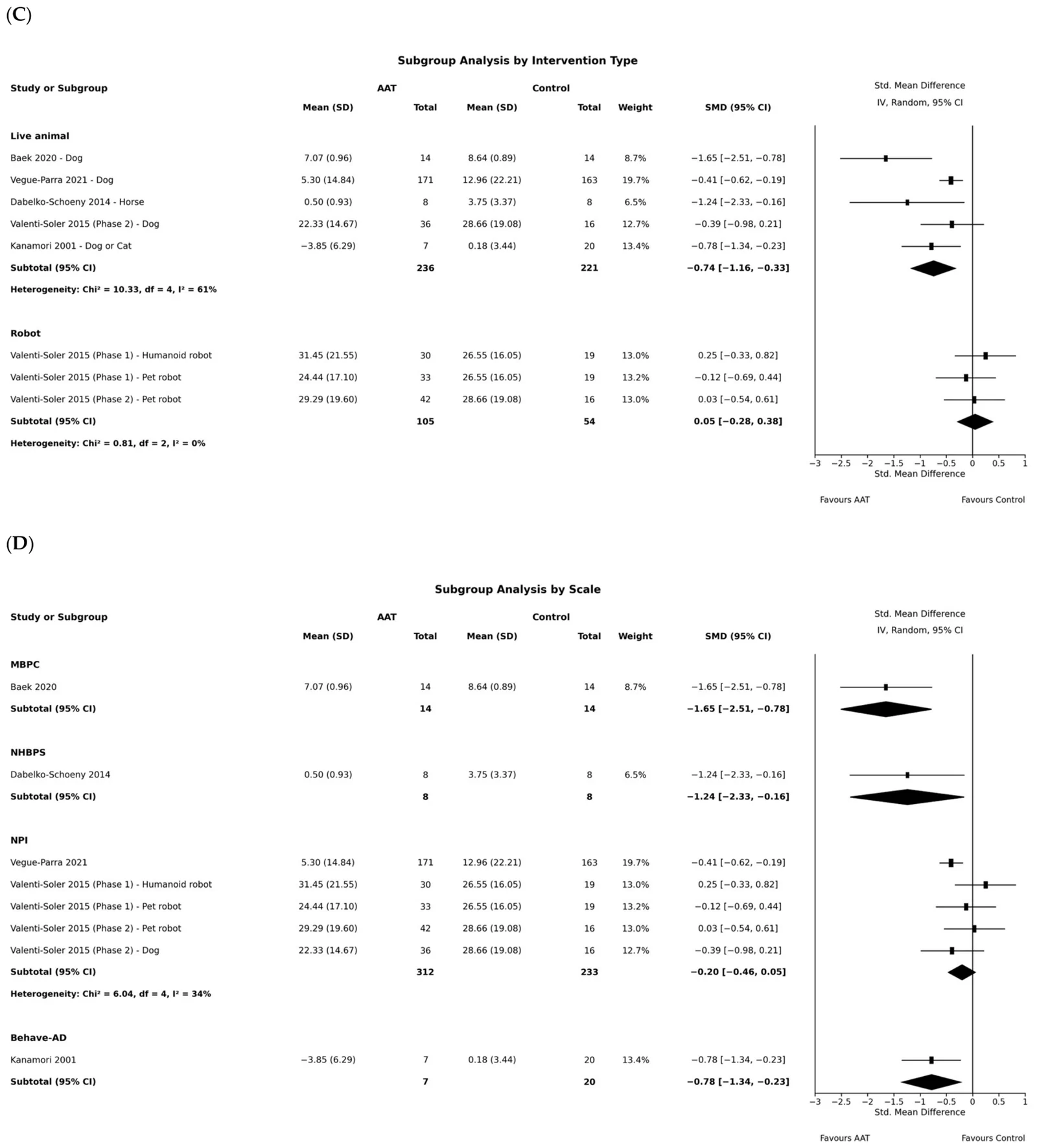
Meta-analysis of overall BPSD outcomes (AAT vs. control): (**A**) random-effects forest plot of standardized mean differences (SMDs); (**B**) leave-one-out influence analysis; (**C**) subgroup analysis by intervention type (live animal vs. robot); (**D**) subgroup analysis by outcome scale [10,11,14,25,28].

In random-effects meta-analysis, AAT was associated with lower overall BPSD severity compared with controls (SMD = −0.53, 95% CI −0.91 to −0.15), with moderate-to-substantial heterogeneity (I^2^ = 73%) (Figure 2A). Leave-one-out analyses demonstrated consistent directionality, with the largest influence attributable to Baek et al. [11]. Exclusion of this study attenuated the pooled estimate while remaining statistically significant (SMD = −0.41, 95% CI −0.74 to −0.08) and reduced heterogeneity (I^2^ = 61%) (Figure 2B). When analysis was restricted to randomized controlled trials, the pooled effect was unchanged (5 comparisons [10,11,14,25]; SMD = −0.50, 95% CI −0.94 to −0.07; I^2^ = 77%). After exclusion of studies at high or serious risk of bias [11,14,28], AAT was no longer associated with a significant reduction in BPSD severity (SMD −0.16, 95% CI −0.38 to 0.07; I^2^ = 0%).

Subgroup analysis by intervention type showed that live animal-assisted interventions were associated with reduced BPSD severity (SMD = −0.74, 95% CI −1.16 to −0.33; I^2^ = 61%), whereas robot-assisted intervention showed no benefit (SMD = 0.05, 95% CI −0.28 to 0.38) (Figure 2C). Scale-based subgroup analyses were limited by single-study contributions for MBPC, NHBPS, and Behave-AD (Figure 2D).

#### 3.5.1. Depressive Symptoms

**Figure 3:**
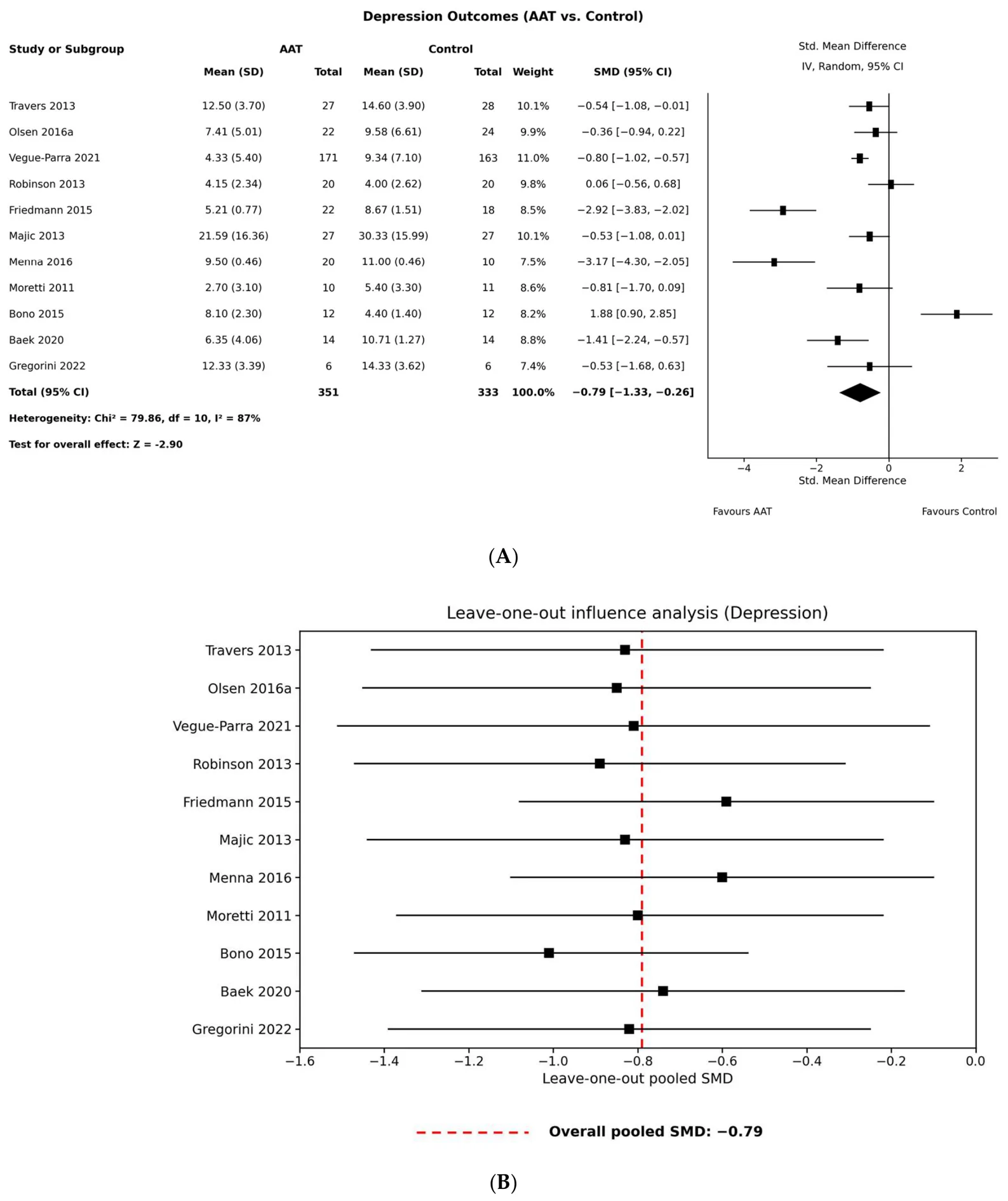

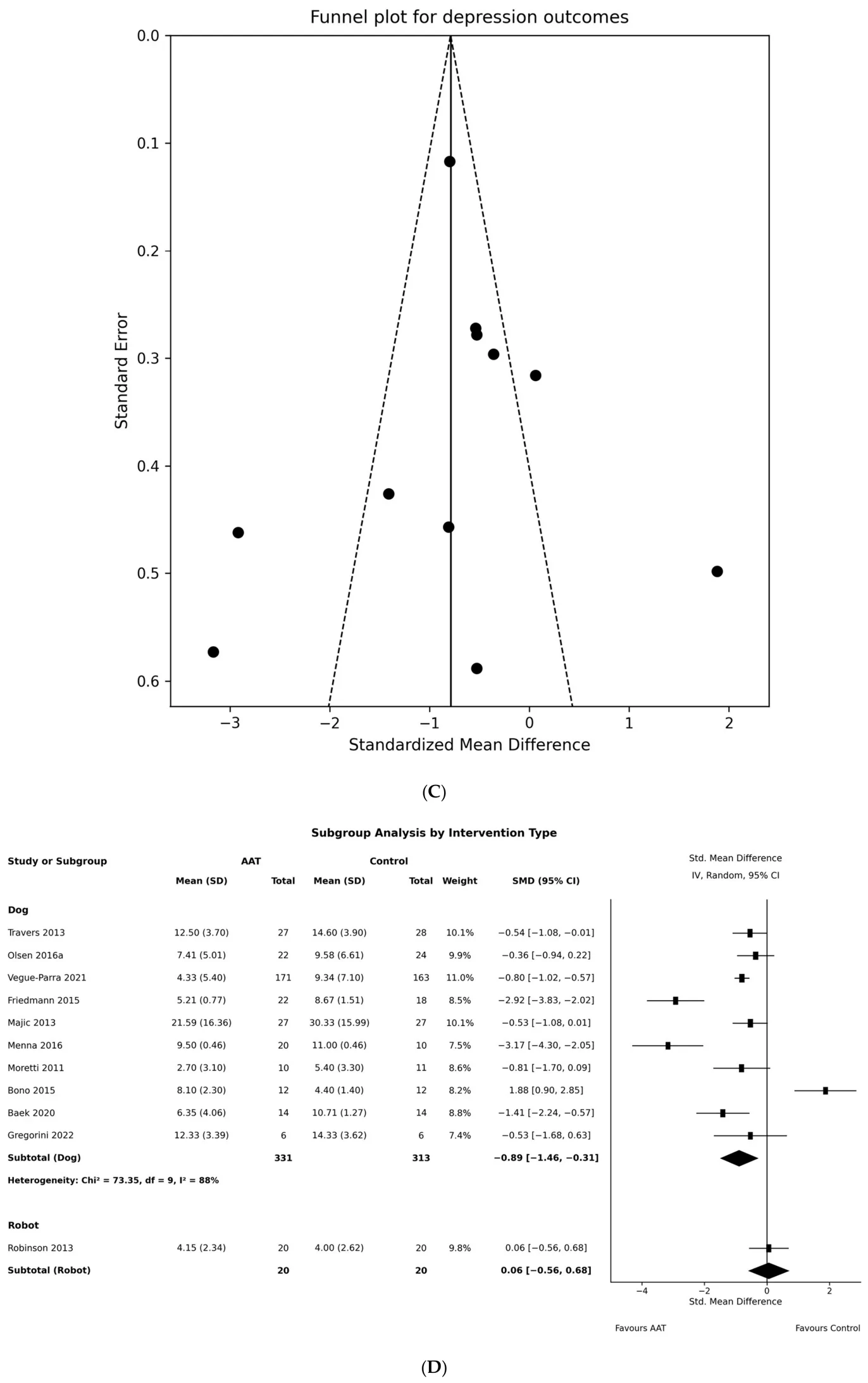

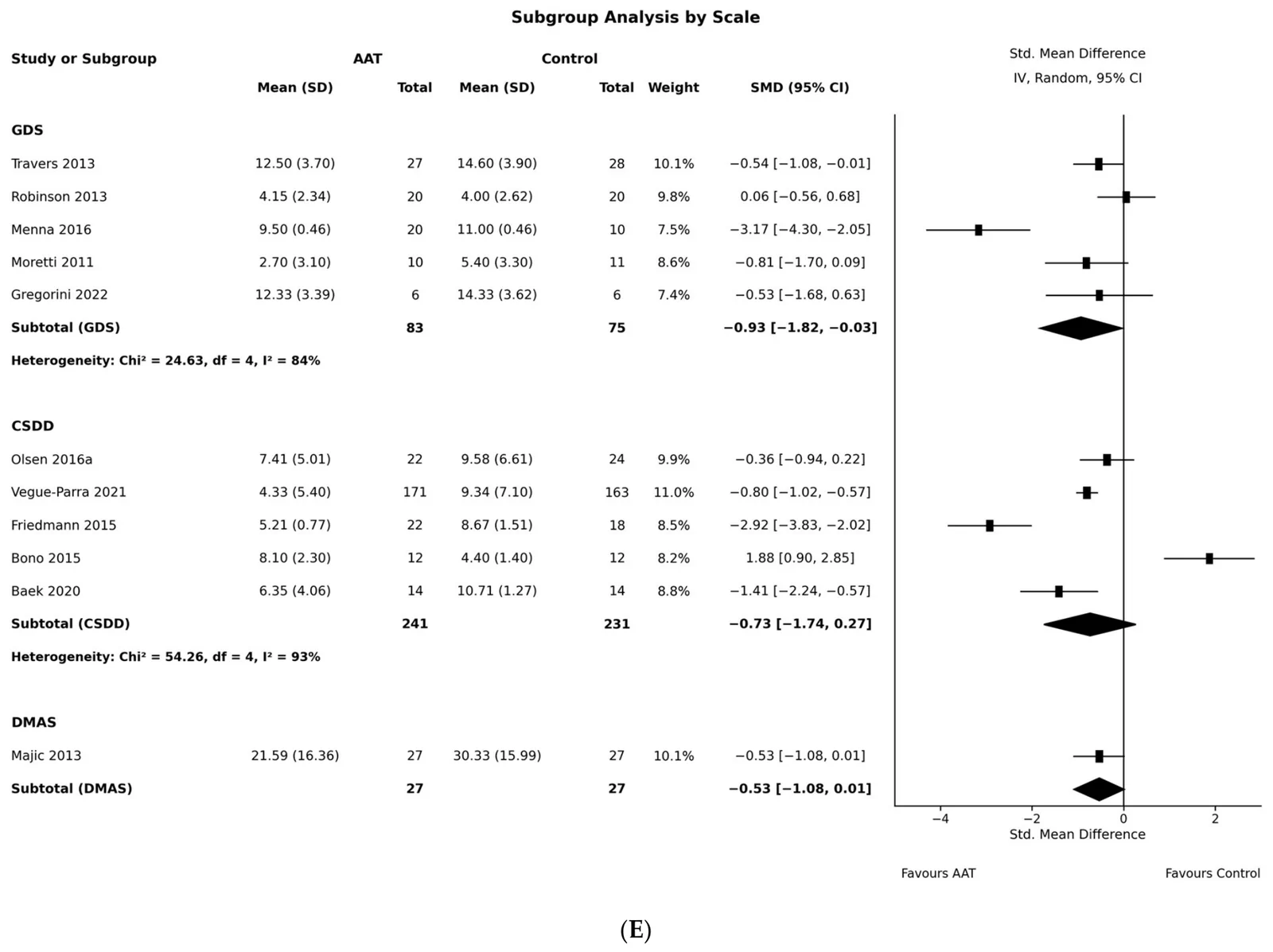
Meta-analysis of depression outcomes (AAT vs. control): (**A**) random-effects forest plot of SMDs; (**B**) leave-one-out influence analysis; (**C**) funnel plot for assessment of publication bias; (**D**) subgroup analysis by intervention type; (**E**) subgroup analysis by depression scale [11,12,15,16,18,22,24,25,27,30,31].

Eleven controlled studies (351 AAT; 333 control) evaluated depressive symptoms using the Geriatric Depression Scale (GDS; *n* = 5), Cornell Scale for Depression in Dementia (CSDD; *n* = 5), and Dementia Mood Assessment Scale (DMAS; *n* = 1). In random-effects meta-analysis, AAT was associated with lower depressive symptom severity compared with control conditions (SMD = −0.79, 95% CI −1.33 to −0.26), with substantial heterogeneity (I^2^ = 87%) (Figure 3A). Leave-one-out influence analysis showed that exclusion of individual studies did not reverse the direction of the pooled effect (Figure 3B). The greatest attenuation of the pooled estimate was observed with the exclusion of Friedmann et al. (SMD = −0.60, 95% CI −1.13 to −0.07; I^2^ = 85.2%) [15] and Menna et al. (SMD = −0.61, 95% CI −1.14 to −0.07; I^2^ = 86.6%) [30]. Exclusion of Bono et al. [12], which reported an effect in the opposite direction, increased the pooled magnitude (SMD = −0.99, 95% CI −1.42 to −0.57; I^2^ = 84.1%). When these three most influential studies were excluded, the pooled effect remained statistically significant with reduced heterogeneity (SMD = −0.60, 95% CI −0.86 to −0.34; I^2^ = 39.9%). When analysis was restricted to randomized controlled trials, the pooled effect remained statistically significant (8 studies [11,12,15,16,18,22,24,25]; SMD = −0.58, 95% CI −1.17 to −0.01; I^2^ = 89%). Exclusion of high-risk studies [11,18] did not eliminate AAT statistical significance (SMD −0.63, 95% CI −1.11 to −0.14, I^2^ = 82%).

Regarding publication bias, visual inspection of the funnel plot showed mild asymmetry (Figure 3C). However, Egger’s regression test did not indicate statistically significant small-study effects (intercept −0.32, 95% CI −3.73 to 3.10), suggesting that the observed asymmetry is more likely driven by substantial between-study heterogeneity rather than publication bias.

Subgroup analysis by intervention type showed a significant association for dog-assisted interventions (SMD = −0.89, 95% CI −1.46 to −0.31; I^2^ = 88%), whereas the single robotic-intervention study showed no evidence of effect (SMD = 0.06, 95% CI −0.56 to 0.68) (Figure 3D). Stratification by depression scale yielded pooled effects of −0.93 for GDS-based studies (95% CI −1.82 to −0.03; I^2^ = 84%) and −0.73 for CSDD-based studies (95% CI −1.74 to 0.27; I^2^ = 93%); the single DMAS study reported SMD = −0.53 (95% CI −1.08 to 0.01) (Figure 3E).

#### 3.5.2. Cognition

**Figure 4:**
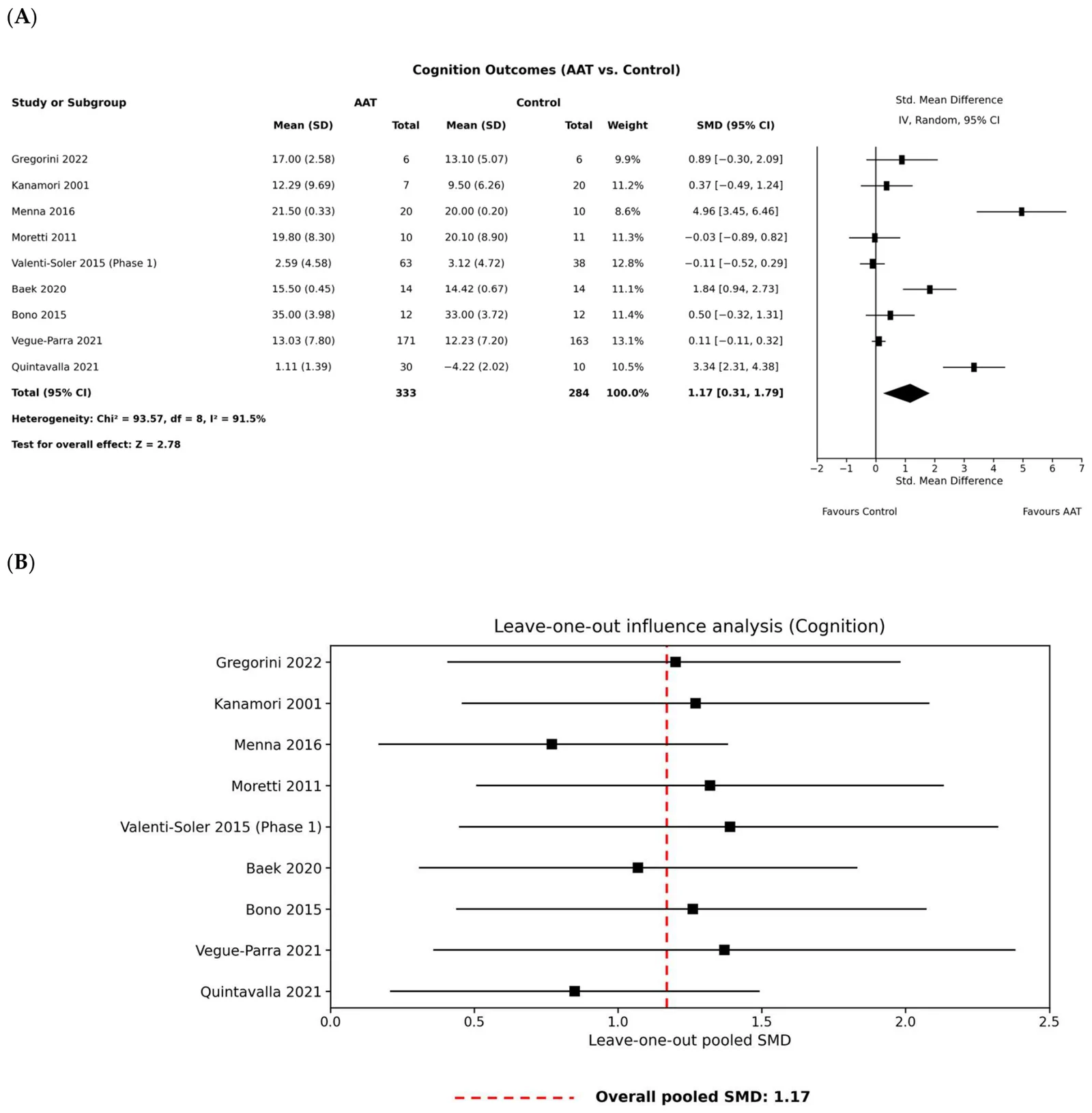
Meta-analysis of cognition outcomes (AAT vs. control): (**A**) random-effects forest plot of SMDs; (**B**) leave-one-out influence analysis [10,11,12,21,25,27,28,30,31].

Nine controlled studies (333 AAT; 284 control) evaluated cognitive performance using the Mini-Mental State Examination (MMSE; *n* = 8) and the Alzheimer’s Disease Assessment Scale—Cognitive Subscale (ADAS-Cog; *n* = 1). AAT was associated with improved cognitive performance compared with controls (SMD = 1.17, 95% CI 0.31 to 1.79), with substantial heterogeneity (I^2^ = 91.5%) (Figure 4A). Leave-one-out analyses demonstrated consistent directionality of effects (Figure 4B). Menna et al. [30] and Quintavalla et al. [21] exerted the greatest influence. Exclusion of either study reduced heterogeneity while preserving statistical significance (Menna et al. [30] excluded: SMD = 0.78, 95% CI 0.22 to 1.35; I^2^ = 63%; Quintavalla et al. [21] excluded: SMD = 0.85, 95% CI 0.28 to 1.42; I^2^ = 69%). When both were excluded, the pooled estimate remained statistically significant with lower heterogeneity (SMD = 0.52, 95% CI 0.18 to 0.86; I^2^ = 41%). When analysis was restricted to randomized controlled trials, the pooled effect remained statistically significant (5 studies [10,11,12,21,25]; SMD = 1.02, 95% CI 0.17 to 1.88; I^2^ = 93%). After exclusion of studies at high or serious risk of bias [11,28], AAT remained associated with improved cognitive outcomes (SMD 1.17, 95% CI 0.18 to 1.91) and substantial heterogeneity (I^2^ = 92.6%).

Scale-based subgroup analysis was not performed due to a single ADAS-Cog study. Exploratory analysis indicated no cognitive benefit for robot-assisted interventions based on a single study with two independent robot arms (SMD = −0.11, 95% CI −0.52 to 0.29).

#### 3.5.3. Agitation

Five controlled studies (129 AAT; 127 control) evaluated agitation using the Cohen–Mansfield Agitation Inventory (*n* = 3), OASS (*n* = 1), and BARS (*n* = 1). AAT was not associated with a statistically significant reduction in agitation compared with control conditions (SMD = −0.81, 95% CI −1.68 to 0.06), with considerable heterogeneity (I^2^ = 90%) (Supplementary Figure S2A). When analysis was restricted to randomized controlled trials, the pooled effect remained non-significant (4 studies [15,16,18,26]; SMD = −0.96, 95% CI −2.03 to 0.12; I^2^ = 93%). Given the small number of studies, further subgroup analyses were not performed.

#### 3.5.4. Quality of Life

Eight independent controlled comparisons from six publications (187 AAT; 139 control) evaluated quality of life using the Quality of Life in Alzheimer’s Disease scale (QoL-AD; five comparisons) and the QUALID scale (three comparisons). One publication [24] contributed three independent facility-level comparisons analyzed separately due to non-overlapping participants. The pooled effect was not statistically significant (SMD = 0.12, 95% CI −0.26 to 0.50), with moderate heterogeneity (I^2^ = 60%) (Supplementary Figure S2B). Seven comparisons reported no statistically significant between-group differences, while one study [13] favored AAT and represented an outlier. Exclusion of this study reduced heterogeneity (I^2^ = 49%). All eight comparisons contributing to this outcome derived from randomized controlled trials [10,13,18,19,22,24], so no restricted analysis was required.

#### 3.5.5. Activities of Daily Living

Five controlled studies (220 AAT; 227 control) assessed functional outcomes using the Barthel Index (*n* = 3) and other ADL scales (*n* = 2). AAT was not associated with a statistically significant improvement in ADL performance (SMD = 0.66, 95% CI −0.18 to 1.50), with substantial heterogeneity (I^2^ = 88.2%) (Supplementary Figure S2C). Leave-one-out analyses did not identify a single influential trial driving the pooled estimate, with confidence intervals crossing the null in all iterations. When analysis was restricted to randomized controlled trials, the pooled effect remained non-significant (4 studies [11,12,13,25]; SMD = 0.84, 95% CI −0.21 to 1.89; I^2^ = 92%).

### 3.6. Certainty of Evidence

Certainty was rated very low for overall BPSD severity, depressive symptoms, cognitive performance, agitation, and activities of daily living and low for quality of life (Supplementary Table S5). All outcomes were rated down for risk of bias, as no included study was judged at low risk. Five outcomes were additionally rated down for inconsistency and five for imprecision.

### 3.7. Controlled Outcomes Not Included in Meta-Analysis

Seven controlled studies evaluated outcomes related to social engagement, motivation, and anxiety using heterogeneous measures that were not amenable to quantitative pooling. Three studies assessed global social or motivational constructs using standardized scales. Observed well-being, measured with the Behavioral Assessment of Care (BAC), was higher in the dog-assisted group compared with the control (103.54 ± 3.21, *n* = 30 vs. 90.62 ± 4.68, *n* = 10; 21). Loneliness, assessed using the UCLA Loneliness Scale, increased in controls but decreased in the intervention group (change +2.22 vs. −4.21; *n* = 20 per group) [22]. In contrast, apathy measured with the Apathy Evaluation Scale showed no evidence of benefit, with higher end-of-study apathy scores in the dog-assisted group than in the control (17.53 ± 0.90 vs. 15.72 ± 0.82) [15].

Four additional controlled studies examined session-level social behavior, affective expression, or physiologic engagement using direct observation or objective monitoring. Using structured behavioral coding, Wesenberg et al. [33] reported greater observed pleasure during AAT compared with control, while negative emotional expressions remained rare in both conditions. Schuurmans et al. [23] found that both dog and robot interventions increased observed engagement compared with handler-only control, with greater overall social interaction in the dog group than the robot group, indicating an amplified social effect of live animals. Similarly, in a controlled clinical trial comparing identical physiotherapy and social stimulation programs with and without a therapy dog, Rodrigo-Claverol et al. [32] demonstrated significantly greater improvement in communication during AAT.

In robotic-companion trials, Petersen et al. [20] reported greater reductions in anxiety in the intervention group compared with control, while Moyle et al. [17] observed lower daytime and nighttime motor activity compared with usual care, consistent with reduced arousal or restlessness during the intervention period.

## 4. Discussion

This systematic review and meta-analysis indicated that AAT was associated with reductions in overall BPSD among individuals with dementia. However, this effect was sensitive to study quality and lost statistical significance when high-bias trials were excluded. Our results also showed that ATT is associated with clinically meaningful benefits in the domains of cognition and mood. These effects remained statistically significant after exclusion of studies at high risk of bias, supporting the robustness of the findings for these outcomes. No significant effects were observed for agitation, quality of life, or functional independence, suggesting that while AAT may enhance psychological and cognitive engagement, its impact on broader functional outcomes remains uncertain.

The observed improvements in depressive symptoms and cognitive performance suggest a potential interaction between affective state and cognitive function in dementia. Depression in dementia may reduce engagement, motivation, and attentional capacity, thereby amplifying apparent cognitive impairment [34]. The antidepressant effects associated with AAT may reflect attachment-related processes, as interaction with a live animal provides a consistent and emotionally responsive presence that does not rely on complex verbal communication [35,36]. This may improve mood, reduce anxiety, and enhance engagement during cognitive tasks. The cognitive benefits are also consistent with arousal-based models of performance, in which increased sensory stimulation enhances attentional engagement [37,38]. Interaction with a live animal provides multimodal input, including tactile, visual, and auditory stimulation, which may activate preserved subcortical arousal systems such as the locus coeruleus–noradrenergic pathway and frontoparietal attentional networks [39,40,41]. By enhancing alertness and attentional control, AAT may allow individuals to perform closer to their existing cognitive capacity during testing.

Another finding of this review is the divergence in outcomes between live animal interventions and robotic animal companions. Live animals reduced depressive symptoms and improved cognition, whereas robotic companions did not show comparable effects. The therapeutic impact likely depends on reciprocal and spontaneous interaction with a living being, which current robotic systems may not fully replicate. The effectiveness of social robots may also be constrained by reduced emotional authenticity or discomfort elicited by imperfect anthropomorphism [42]. One contributing mechanism may be the perception of biological motion. Movement generated by living beings carries kinematic regularities that are detected independently of body form or viewpoint [43], and such motion engages cerebellar circuits connected with fronto-parietal networks [44,45]. Robotic companions resemble animals in form, but their movement is actuator-driven and may not carry these features. Nonetheless, conclusions regarding robotic interventions should be interpreted with caution. Only a few controlled studies contributed to the robotic subgroup for depression and cognition, and the associated confidence intervals were wide enough to include effects comparable to those observed with live animals. The absence of a detected effect therefore cannot be distinguished from a Type II error and should not be taken as evidence that robotic companions are ineffective. Additionally, individual robotic-companion trials reported domain-specific benefits, including reductions in anxiety and restlessness [17,20], suggesting that robotic interventions may influence arousal-related or behavioral outcomes even in the absence of broader mood or cognitive effects.

The differential response observed between depressive symptoms and agitation may reflect partially distinct neurobiological underlying mechanisms. Agitation in Alzheimer’s disease has been conceptualized as arising from disruption of frontal–subcortical regulatory circuits, resulting in impaired executive control over limbic reactivity [46]. This disruption is frequently associated with behavioral disinhibition and progressive structural and neurochemical changes that impair inhibitory regulation, potentially contributing to greater variability in response to single-domain psychosocial interventions. In contrast, depressive symptoms in dementia are closely linked to fronto-limbic and motivational networks that may be more amenable to modulation through social and affective engagement [47]. Consistent with this distinction, prior systematic review of non-pharmacological interventions has reported more robust benefits for mood-related outcomes compared to agitation, where effects are frequently small or inconsistent across intervention types [48].

The lack of significant effects on ADL and quality of life must be viewed within the context of progressive neurodegeneration. Interventions targeting affect cannot reverse established functional impairment. Furthermore, null QoL findings may reflect measurement limitations, as proxy-rated instruments often lack the sensitivity to capture transient improvements in well-being [49,50].

These findings have practical implications for dementia care. The interventions reviewed were delivered in nursing homes, assisted living facilities, day centers, and geriatric wards, settings where people with dementia already receive care, and the benefits observed fell under domains for which pharmacological options are limited. Implementation would require trained therapy animals and handlers, structured protocols delivered over sustained periods, and institutional provision for animal welfare, infection control, allergy screening, and resident consent. Against these requirements, AAT is inexpensive relative to pharmacological alternatives and imposes no medication burden. Economic and implementation data are absent from this literature and would be needed before service-level recommendations could be made.

### Limitations and Future Directions

Several limitations affect these findings. First, the overall methodological quality of the included studies was limited. None of the controlled studies were judged to be at low risk of bias. This raises concern that some observed effects may be inflated due to performance bias, lack of blinding, incomplete outcome reporting, or small sample sizes. Future trials should incorporate pre-registered protocols, transparent allocation concealment, and adherence to CONSORT reporting standards to improve internal validity.

Second, the number of studies contributing to several outcome domains was small, limiting statistical power and resulting in unstable sensitivity analyses. For instance, the loss of statistical significance for total BPSD after exclusion of high-risk trials reflected reliance on only three comparisons from two moderate-risk studies. Additionally, the absence of a detected benefit for robotic interventions could be attributed to limited statistical power rather than to demonstrated ineffectiveness, and adequately powered trials of robotic companions are needed before the two intervention types can be meaningfully compared. Larger, adequately powered multicenter randomized controlled trials are needed to generate more stable effect estimates. Third, outcomes within each domain were measured with different instruments, which vary in construct and in how they weight constituent symptoms. Fourth, no study reported progression or conversion endpoints, so the effect of AAT on the trajectory of cognitive decline could not be assessed.

Fifth, the mechanistic basis of AAT remains insufficiently characterized. Future research integrating physiological markers of arousal, attentional measures, or neuroimaging approaches may clarify whether observed improvements reflect state-dependent modulation of engagement rather than a structural modification of disease processes. Lastly, heterogeneity was substantial across the main pooled outcomes and was not resolved by sensitivity analyses. This most likely reflects clinical and methodological variation between studies, including differences in baseline dementia severity, dementia subtype, the length and intensity of exposure, and the structure of intervention protocols. These sources could not be examined formally as severity was measured with non-comparable instruments and reported as group means or eligibility ranges rather than severity strata; dementia subtype was inconsistently reported, and intervention protocols differed in ways that made total exposure non-comparable. Additionally, control conditions differed across studies, ranging from usual care to time- or attention-matched active comparators in which participants received structured human contact without an animal. Comparisons against usual care capture the effect of the intervention as delivered, including its social and attentional components, whereas attention-matched comparisons isolate the contribution of the animal. Effect estimates from individual studies are therefore not directly comparable. Meta-regression was not appropriate given the small number of studies contributing to each outcome. The sources of heterogeneity therefore remain unexplained, and pooled estimates should be interpreted with corresponding caution.

## 5. Conclusions

Current evidence supports AAT as a potent non-pharmacological intervention for improving mood and cognitive engagement in individuals with dementia, with more limited or inconsistent effects across other domains. These findings underscore the need for rigorously designed and adequately powered trials to further refine the role of AAT in dementia care.

## Figures and Tables

**Table 1:** Characteristics of included controlled studies.

Study (Country)	Design	N Analyzed (by Group)	Sex (F/M)	Intervention: Type and Dose	Comparator	Outcomes Contributing to This Review
Baek 2020 (South Korea) [11]	RCT (cluster, 2 sites)	28 (14/14)	AAT 4F/10M; control 2F/12M	Live dog; 60 min, 2×/wk, 8 wk	Conventional care	BPSD; depression; cognition; ADL
Bono 2015 (Italy) [12]	RCT	24 (12/12)	8F/4M per group	Live dog; ~60 min, biweekly, 8 mo	Home follow-up, same intervals	Depression; cognition; ADL
Briones 2021 (Spain) [13]	RCT (ball draw)	34 (16/18)	AAT 13F/3M; control 12F/6M	Live dogs; 50 min, weekly, 9 mo	Care as usual	QoL; ADL
Dabelko-Schoeny 2014 (USA) [14]	RCT (crossover)	16 (crossover, all completed)	9F/7M (56.3% F)	Live horses (ground-based); weekly, 4 wk	Treatment as usual	BPSD
Friedmann 2015 (USA) [15]	RCT (cluster)	37 (19/18)	29F/11M overall (PAL 15F/7M; control 14F/4M)	Live dog; 60–90 min, 2×/wk, 12 wk	Reminiscing (attention-matched)	Depression; agitation; apathy (narrative)
Gregorini 2022 (Italy) [27]	Non-randomized controlled (4-group factorial: diagnosis × therapy)	24 enrolled (AD 6/6; non-AD 6/6); AD stratum pooled	18F/6M overall	Live dogs; 45 min, weekly, 10 wk	No AAT; no animal contact	Depression; cognition
Kanamori 2001 (Japan) [28]	Non-randomized controlled	27 (7/20)	AAT 5F/2M; control 16F/4M	Live animals; 6 sessions, biweekly	Day care without AAT	BPSD; cognition; ADL
Krüger 2022 (Germany) [29]	Non-randomized within-subject controlled, investigator-blinded	26 (13/13)	11F/15M (42% F)	Live dog in milieu therapy; ~20 min/day	Same ward physician present without dog (active control)	Agitation
Majić 2013 (Germany) [16]	RCT (matched pairs, random allocation within pair)	54 (27/27)	38F/16M matched sample (70.4% F)	Live dog + TAU; ≤45 min, weekly, 10 wk	Treatment as usual	Depression; agitation
Menna 2016 (Italy) [30]	Non-randomized controlled	50 (AAT 20/ROT 20/control 10)	AAT 16F/4M; ROT 14F/6M; control 7F/3M	Live animal + ROT protocol; 45 min, weekly, 6 mo	ROT only; usual care	Depression; cognition
Moretti 2011 (Italy) [31]	Non-randomized case–control	21 (10/11)	20F/1M (95.2% F)	Live dog; 90 min, weekly, 6 wk	No pet therapy	Depression; cognition
Moyle 2018 (Australia) [17]	RCT (cluster, 28 sites; 3 arms)	415 enrolled; 175 daytime/280 night-time analyzed	Predominantly female (~75–80% across arms)	PARO robot; 15 min, 3×/wk, 10 wk	Plush toy (PARO with robotic features disabled); usual care	Motor activity; sleep (narrative)
Olsen 2016 (Norway) [18]	RCT (cluster)	51 (25/26)	60.0% F (AAT); 65.4% F (control)	Live dog; 30 min, 2×/wk, 12 wk, group	Usual care	Depression; agitation; QoL
Olsen 2016 (Norway) [19]	RCT (cluster)	79 (41/38)	51.2% F (AAT); 60.5% F (control)	Live dog; 30 min, 2×/wk, 12 wk, group	Usual care	QoL
Petersen 2016 (USA) [20]	RCT	61 (35/26)	77% F overall; comparable between arms	PARO robot; 20 min, 3×/wk, 12 wk, group	Usual care	Anxiety (narrative)
Quintavalla 2021 (Italy) [21]	RCT	40 (30/10)	29F/11M overall	Live dog; 30 min, 2×/wk, 12 wk (24 sessions)	No AAT	Cognition; well-being (narrative)
Robinson 2013 (New Zealand) [22]	RCT	40 (20/20)	27F/13M overall	PARO robot; two 1-h group sessions/wk, 12 wk	Normal activities	Depression; QoL; loneliness (narrative)
Rodrigo-Claverol 2020 (Spain) [32]	Non-randomized cluster controlled (allocation by center)	46 (23/23)	35F/11M overall (AAT 69.6% F; control 82.6% F)	Live dog integrated into physiotherapy + social stimulation; weekly, 12 wk	Same program without dog	Communication (narrative)
Schuurmans 2021 (Netherlands) [23]	RCT	47 (dog 19/robot 16/control 12)	46F/20M overall at baseline	Live dog vs. robot pet; weekly group sessions	Handler-only sessions; robot-assisted arm (depending on comparison)	Observed engagement (narrative)
Travers 2013 (Australia) [24]	RCT (multicenter)	55 (27/28)	AAT 19F/8M; control 24F/4M	Live dog; 40–50 min, 11 wk, group; 3×/wk (Facility A), 2×/wk (Facilities B, C)	Human therapist only	Depression; QoL
Valentí Soler 2015—Phase 1 (Spain) [10]	RCT	101 (PARO 33/NAO 30/control 38); robot arms combined vs. control	~88% F overall	NAO or PARO robot; 2×/wk, 3 mo	Standard therapy sessions	BPSD; cognition
Valentí Soler 2015—Phase 2 (Spain) [10]	RCT	110 (PARO 42/dog 36/control 32); intervention arms combined vs. control	~90% F overall	PARO robot or live dog; 2×/wk, 3 mo	Standard therapy sessions	BPSD; QoL
Vegue Parra 2021 (Spain) [25]	RCT	334 (171/163)	259F/75M overall (77.5% F)	Live dog; 45 min, weekly, 8 mo, group	Usual care	BPSD; depression; cognition; ADL
Wesenberg 2019 (Germany) [33]	Non-randomized within-subject controlled	17 completed (within-subject)	13F/4M (~76% F)	Live dog; 45 min, weekly, group	Usual care	Observed pleasure (narrative)
Pope 2016 (USA) [26]	RCT (crossover)	44 (crossover, all enrolled)	20F/24M (45.5% F)	Live dog; 10 min, 2×/wk, 2 wk, crossover	Human interaction	Agitation

## Data Availability

The data supporting the findings of this study are derived from previously published studies, which are cited in the manuscript and its Supplementary Materials. The extracted datasets used in the meta-analyses are available from the corresponding author upon reasonable request.

## Supplementary Materials

The following supporting information can be downloaded at https://www.mdpi.com/article/10.3390/jcm15176654/s1. Table S1: PRISMA Checklist [51]; Table S2: Search strategies; Table S3: Characteristics of non-controlled eligible studies [52,53,54,55,56,57,58,59,60,61,62,63,64,65,66,67,68,69,70,71,72,73,74,75,76,77,78,79,80,81,82,83,84,85,86]; Table S4: Risk-of-bias assessment for controlled studies [10,11,12,13,14,15,16,17,18,19,20,21,22,23,24,25,26,27,28,29,30,31,32,33]; Table S5: GRADE Summary of Findings [10,11,12,13,14,15,16,17,18,19,20,21,22,23,24,25,26,27,28,29,30,31,32,33]; Figure S1: Risk-of-bias summary for (A) randomized controlled trials (RoB 2) and (B) non-randomized controlled trials (ROBINS-I); Figure S2: Forest plots for (A) agitation, (B) quality of life, and (C) activities of daily living outcomes [10,11,12,13,15,16,18,19,22,24,25,26,28,29].
