# Supplementary material for: Animal-Assisted Therapy in Dementia: A Systematic Review and Meta-Analysis of Cognitive, Affective, and Behavioral Outcomes
Source: J Clin Med. 2026 Aug 28;15(17):6654. doi: 10.3390/jcm15176654 (PMC13566663; doi:10.3390/jcm15176654)
Supplement: Supplementary file 1 [file jcm-15-06654-s001.zip › Supplementary_Table_S2.pdf]

Supplementary Table S2. Search Strategies

Medline

1 exp dementia/ or exp Alzheimer Disease/ or exp aids dementia complex/ or exp dementia, vascular/ or exp frontotemporal lobar degeneration/ or exp lewy body disease/ or exp mixed dementias/ or exp Dementia, Multi-Infarct/ or exp Postoperative Cognitive Complications/ 227967
2 (dementia* or senile or encephalopath* or cognitive or cognition or Alzheimer* or lewy body).tw,kf. 885586
3 1 or 2 920918
4 exp Animal Assisted Therapy/ 940
5 ((animal-assisted or pet or pets or animal facilitated or robot or robots) adj3 therap*).tw,kf. 2613
6 Animals/ 7665219
7 assisted therapy.tw,kf. 2053
8 6 and 7 477
9 4 or 5 or 8 3316
10 3 and 9 265
11 limit 10 to english language 244

Embase

1 exp dementia/ or exp HIV associated dementia/ or exp dementia, vascular/ or exp frontotemporal lobar degeneration/ or exp diffuse Lewy body disease/ or exp mixed dementia/ or exp multiinfarct dementia/ or exp postoperative cognitive dysfunction/ or exp Alzheimer disease/ 509421
2 (dementia* or senile or encephalopath* or cognitive or cognition or Alzheimer* or lewy body).tw,kf. 1221804
3 1 or 2 1344828
4 exp animal assisted therapy/ 1810
5 ((animal-assisted or pet or pets or animal facilitated or robot or robots) adj3 therap*).tw,kf. 753
6 animal/2192697
7 assisted therapy.tw,kf. 2753
8 6 and 7 257
9 4 or 5 or 8 2012
10 3 and 9 361
11 limit 10 to english language 343

APA PsycINFO

1 exp dementia/ or exp aids dementia complex/ or exp alzheimer's disease/ or exp dementia with lewy bodies/ or exp frontotemporal lobar degeneration/ or exp presenile dementia/ or exp senile dementia/ or exp vascular dementia/ or exp cognitive impairment/ 135329
2 (dementia* or senile or encephalopath* or cognitive or cognition or Alzheimer* or lewy body).tw. 681381
3 1 or 2 686010
4 exp Animal Assisted Therapy/ 1678
5 ((animal-assisted or pet or pets or animal facilitated or robot or robots) adj3 therap*).tw. 1099
6 animals/ 7609
7 assisted therapy.tw. 1563
8 6 and 7 19
9 4 or 5 or 8 2001
10 3 and 9 302
11 limit 10 to english language 273

CAB Abstract

1 exp dementia/ or exp AIDS dementia complex/ or exp Alzheimer's disease/ 17063
2 (dementia* or senile or encephalopath* or cognitive or cognition or Alzheimer* or lewy body).ti,ab. 79741
3 1 or 2 80126
4 animal assisted therapy/ 781
5 ((animal-assisted or pet or pets or animal facilitated or robot or robots) adj3 therap*).ti,ab. 473
6 animals/ 6937014
7 assisted therapy.ti,ab. 354
8 6 and 7 332
9 4 or 5 or 8 1081
10 3 and 9 99
11 limit 10 to english language 80

Scopus

(INDEXTERMS (dementia OR "Alzheimer Disease" OR "aids dementia complex" OR "HIV associated dementia" OR "dementia, vascular" OR "frontotemporal lobar degeneration" OR

"lewy body disease" OR "diffuse Lewy body disease" OR "mixed dementias" OR "mixed dementia" OR "Dementia, Multi-Infarct" OR "multiinfarct dementia" OR "Postoperative Cognitive Complications" OR "postoperative cognitive dysfunction") OR TITLE-ABS-KEY (dementia* OR senile OR encephalopath* OR cognitive OR cognition OR alzheimer* OR "lewy body")) AND (INDEXTERMS ("Animal Assisted Therapy") OR TITLE-ABS-KEY ((("animal-assisted" OR "animal assisted" OR "pet" OR "pets" OR "animal facilitated" OR "robot" OR "robots") W/3 therap*))) AND (LIMIT-TO (LANGUAGE,"English"))

Web of Science

TS=((dementia* OR senile OR encephalopath* OR cognitive OR cognition OR Alzheimer* OR "lewy body") AND ((("animal-assisted" OR "pet" OR "pets" OR "animal facilitated" OR "robot" OR "robots") NEAR/3 therap*)))

Cochrane CENTRAL (trials)

ID	Search	Hits
#1	MeSH descriptor: [Dementia] explode all trees	9898
#2	MeSH descriptor: [Alzheimer Disease] explode all trees	5593
#3	MeSH descriptor: [AIDS Dementia Complex] explode all trees	95
#4	MeSH descriptor: [Dementia, Vascular] explode all trees	459
#5	MeSH descriptor: [Frontotemporal Lobar Degeneration] explode all trees	143
#6	MeSH descriptor: [Lewy Body Disease] explode all trees	165
#7	MeSH descriptor: [Mixed Dementias] explode all trees	4
#8	MeSH descriptor: [Dementia, Multi-Infarct] explode all trees	77
#9	MeSH descriptor: [Postoperative Cognitive Complications] explode all trees	142
#10	((dementia* OR senile OR encephalopath* OR cognitive OR cognition OR Alzheimer* OR "lewy body")):ti,ab,kw	133853
#11	#1 OR #2 OR #3 OR #4 OR #5 OR #6 OR #7 OR #8 OR #9 OR #10	134118
#12	MeSH descriptor: [Animal Assisted Therapy] explode all trees	152
#13	((("animal-assisted" OR pet OR pets OR "animal facilitated" OR "robot" OR "robots") NEAR/3 therap*)):ti,ab,kw	476
#14	#12 OR #13	551
#15	#11 AND #14	79
