# Supplementary material for: Animal-Assisted Therapy in Dementia: A Systematic Review and Meta-Analysis of Cognitive, Affective, and Behavioral Outcomes
Source: J Clin Med. 2026 Aug 28;15(17):6654. doi: 10.3390/jcm15176654 (PMC13566663; doi:10.3390/jcm15176654)
Supplement: Supplementary file 1 [file jcm-15-06654-s001.zip › Supplementary_Table_S3.pdf]

Supplementary Table S3. Characteristics of non-controlled eligible studies

Study	Country	Setting	Design	N	Dementia type/severity	Intervention (animal/modality)	Dose	Comparator
Batson 1998 [51]	USA	Long-term care (special care units)	Controlled crossover (within-subjects)	25 (22 analyzed)	Alzheimer's, Moderate	Dog (Live animal)	1 × 10-min session (crossover)	None (uncontrolled)
Belanger 2023 [52]	USA	Community-based setting in Southwest Florida	Pre–post (single arm)	6 persons with dementia and 6 care partners	Not specified; ACL score of 3.8	Dog (Live animal)	Once per week | 90 minutes per session, over 6 consecutive weeks	None (uncontrolled)
Borges de Araujo 2019[53]	Brazil	Biomechanics Laboratory of a local university	Pre–post (single arm)	10 (9 analyzed)	Alzheimer's; Mild to moderate (Clinical Dementia Rating 0.5, 1 or 2)	Horse (Live animal)	Twice Weekly Sessions lasting 30 mins each | 10 weeks	None (uncontrolled)
Edwards 2002 [54]	USA	Dementia-specific extended-care units in three long-term care facilities in Indiana, USA	Pre–post (single arm)	62	Alzheimer's; Not specified	Fish in aquarium (Live animal)	Continuous presence at every meal | 2-week intensive exposure	None (uncontrolled)
Edwards 2014 [55]	USA	Three specialized dementia units	Pre-Post (Single arm)	72	Not specified	Fish in aquarium (Live animal)	Present constantly | 10 weeks	None (uncontrolled)
Fields 2018 [56]	USA	The study took place at two Northern Colorado sites: a for-profit memory care facility off	Mixed Method Case Study	8	Not Specified	Horse (Live animal)	once a week | 8 weeks	None (uncontrolled)
Fogelson 2022 [57]	USA	60-bed residential care facility (ALMC, MC, LTC) in the northeastern US	Pre–post (single arm)	18	Not specified	Robot pet (Robotic/animal-like)	Companion pet continuous | 6 weeks	None (uncontrolled)
Friedmann 2019 [58]	USA	Four small assisted-living facilities (4–9 residents per session) in the U	Secondary longitudinal data analysis	22	Not specified; Mild to moderate cognitive impairment	Dog (Live animal)	2 sessions/week for 12 weeks (total 24)** | Semi-structured 60–90 minutes (M = 64.59 ± 2.96 min)	None (uncontrolled)
Greer 2001[59]	USA	Bethany Homes nursing facility (long-term care unit)	Randomized trial	6	Not specified; Moderate	Live Cat	3 10 minute sessions | 3 10 minute sessions	Toy Cat
Gustafsson 2015 [60]	Sweden	Dementia care home situated in a medium-sized	single case study, and qualitative	4 for quantitative n=14 for qualitative	Not specified	Robotic Cat	free access | 12 weeks	None (uncontrolled)

		municipality in the middle of Sweden	study of caregivers					
Jones 2018 [61]	Australia	permanent residents of a facility within a 100km radius of Brisbane's Central Bus	Pre-Post (Single arm)	138	Alzheimer's disease: 49 (36 %) Vascular dementia: 17 (12 %) Dementia with Lewy bodies: 1 (1 %) Fronto-temporal lobar degeneration: 4 (3 %) Alcohol-related dementia: 1 (1 %) Unspecified: 66 (48 %) ; RUDAS total score: 6.5 ± 6.5	Robot pet (Robotic/animal-like)	3 15-min afternoon sessions a week | 10 weeks	None (uncontrolled)
Karefjard 2019 [62]	Sweden	Three municipal nursing homes in a medium-sized Swedish town	Pre-Post-test (Single arm)	59	Alzheimer's disease: 14 (23.7%)l Vascular dementia: 12 (20.3%); Frontotemporal dementia: 1 (1.7%)' Dementia NS: 24 (40.7%); Alcohol-related dementia: 2 (3.4%); Mixed dementia: 6 (10.2%); All participants had moderate to severe dementia (per inclusion)	Dog (Live animal)	20-45 min one-on-one sessions twice a week, but sometimes only once a week | 10 sessions	None (uncontrolled)
Kawamura 2007 [63]	Japan	residential nursing home in a large city in northern Japan	Pre-Post-test (Single arm)	10	Six subjects had vascular dementia , and four had senile dementia; Not specified	Dog (Live animal)	twice a month for 2 hours, participants played with dogs for 30 mins | 12 months	None (uncontrolled)
Kongable 1989 [64]	USA	20-bed special care Alzheimer's unit, large midwestern Veterans' Home	Pre-Post-test (Single arm)	12	Not specified	Dog (Live animal)	1 day/week | Sessions lasted 3 hours each visit	None (uncontrolled)
Kramer 2009 [65]	USA	Dementia care home	Repeated-measures, within-subjects observational study	18	Not specified	Dog (Live animal)	1 visit a week | 3 weeks	Robotic Dog
LaRose 2022[66]	USA	A community-based adult day	Pre–post (single arm)	12	Not Specified	Robotic Cat	4 DAYS A WEEK | 12 visits, with a	None (uncontrolled)

		center (ADC) in Texas					duration of 30 minutes for each session	
Lassell 2021 [67]	USA	Adaptive riding took place in an indoor arena	descriptive case study design	8	Posterior cortical atrophy (n = 1), Early-onset (n = 1), Mild cognitive impairment (n = 1), Alzheimer's (n = 1), Not specified (n = 4); 2 early–moderate; 2 moderate–later	Horse (Live animal)	Weekly sessions | 1-hour sessions over eight weeks	None (uncontrolled)
Latoch 2022[68]	France	Special care unit (SCU)	Crossover	26	Alzheimer disease 16 61.54% Frontotemporal dementia 4 15.39% Mixed dementia 3 11.54% Vascular dementia 2 7.69% Progressive supranuclear palsy; MMSE score was 6.85 ± 6.87.	Dog (Live animal)	Constant Presence + Animal mediation sessions for 60 mins<br>Present for month 1 , removed for month 2, re-introduced in month 3 (CM2) | 3 Months	None (uncontrolled)
Libin 2004 [69]	USA	Special care unit of a large, suburban, not-for-profit nursing home	Crossover	9	Primary degenerative dementia; Not specified	Robotic Cat	2 session | 10 minutes	None (uncontrolled)
Marx 2010 [70]	USA	2 suburban Maryland nursing homes, all with a diagnosis of dementia	Pre–post (Four arms)	56	Not specified	Dog (Live animal)	1 trial per participant | 15 min	Robotic dog, Puppy video, Plush dog
McCabe 2002 [71]	USA	22-bed SCU of an urban extended health care facility in a midwestern state	Within-participants repeated-measures design	22	probable Alzheimer's disease or related disorder; Not reported	Dog (Live animal)	Daily presence on the unit | 4-week intervention period	None (uncontrolled)
Mossello 2011 [72]	Italy	Alzheimer Day Care Center	Within-participants repeated-measures design	10	Not specified	Robotic dog	3 days/week | 100 minutes/session (10:00–12:00)	None (uncontrolled)
Motomura 2004 [73]	Japan	Local nursing home	Pre–post (single arm)	8	Four patients had dementia of Alzheimer's type (DAT), and the others had vascular dementia; very mild cognitive change	Dog (Live animal)	Over four consecutive days | 1 hour per day	None (uncontrolled)

Naganuma 2015 [74]	Japan	Nursing home	Trial experiments	Not specified	Not specified	Robotic pet	Not specified	None (uncontrolled)
Nordgren 2012 [75]	Sweden	nursing home	Case-report	1	Not specified	Dog (Live animal)	60 mins once a week | 8 weeks	None (uncontrolled)
Nordgren 2014 [76]	Sweden	Eight nursing homes in Eskilstuna municipality, Sweden (four homes already using AAI	Pre–post (single arm)	20	Not specified	Dog (Live animal)	Sessions once or twice per week | 10 sessions, each session 45–60 minutes	None (uncontrolled)
Perez-Saez 2020 [77]	Spain	National Reference Centre for Alzheimer’s and Dementia care	A-B-A-B withdrawal single-case experimental design	3	Not specified	Dog (Live animal)	5 consecutive daily sessions per phase | 15 minutes each	None (uncontrolled)
Richeson 2003 [78]	USA	New England nursing home	Pre–post (single arm)	17 (15 analyzed)	Not specified	Dog (Live animal)	Daily sessions, Monday through Friday.1 hour | 1 hour/day, 5 days/week, for 3 weeks.	None (uncontrolled)
Sanchez-Valdeon 2019 [79]	Spain	Alzheimer’s day care center	Non-randomized controlled	10	Alzheimer’s; Mild to moderate	Dog (Live animal)	weekly individual sessions | 30 minutes per session	None (uncontrolled)
Santaniello 2020 [80]	Italy	Two Alzheimer’s Centers	Pre–post (single arm)	65	Alzheimer’s, Mild to moderate	Dog (Live animal)	Weekly sessions, with a total duration of 45 min per session, for a total period of 6 months	None (uncontrolled)
Sellers 2006 [81]	USA	One skilled nursing / long-term care facility in a rural community	A-B-A-B withdrawal single-case experimental design	4	Not specified	Dog (Live animal)	4 phases | 28 days	None (uncontrolled)
Shawish 2024 [82]	Jordan	Patient's homes	case study	1	Alzheimer’s	Cat (Live animal)	Not specified	None (uncontrolled)
Tamura 2004 [83]	Japan	Geriatric health care facility	Pre–post (single arm)	13		Robotic dog	once | 4 days (5 min each day)	None (uncontrolled)
Thunberg 2020 [84]	Sweden	residential home	Qualitative study with thematic analysis	Cat: eight Dog: two	Ann had vascular dementia and Jacob had Alzheimer's disease; Not specified`	Robotic dog/ cat	Not mentioned clearly | nine-month period	None (uncontrolled)
Tournier 2017 [85]	France	French nursing home's care unit for residents	Pre–post (single arm)	15 (11 after withdrawals)	Alzheimer's disease or other types of senile dementia; Mini-Mental State Examination (MMSE) score was	Dog (Live animal)	Once a week | 5 months (19 sessions)	None (uncontrolled)

					7.8 out of 30, indicating severe dementia. 73% of participants were diagnosed with severe dementia, and the rest were diagnosed with moderately severe dementia			
