# Supplementary material for: Animal-Assisted Therapy in Dementia: A Systematic Review and Meta-Analysis of Cognitive, Affective, and Behavioral Outcomes
Source: J Clin Med. 2026 Aug 28;15(17):6654. doi: 10.3390/jcm15176654 (PMC13566663; doi:10.3390/jcm15176654)
Supplement: Supplementary file 1 [file jcm-15-06654-s001.zip › Supplementary_Table_S4.pdf]

Supplementary Table S4. Risk of bias judgement for controlled studies

Baek 2020.

RoB 2 domain	Judgment	Justification
**Bias arising from the randomization process**	**High risk**	Although cluster allocation was performed using a coin toss at the hospital level, only two clusters were included (one per group). Participants were recruited after cluster assignment, and substantial baseline imbalances were present between groups in cognitive function, emotional status, and activities of daily living, indicating a high risk of selection bias inherent to the randomization process.
**Bias due to deviations from intended interventions**	**Some concerns**	Blinding of participants and care providers was not feasible due to the nature of the animal-assisted intervention. While cluster allocation was intended to reduce contamination, the study did not report procedures to monitor adherence or prevent differential co-interventions beyond routine care, and analyses accounting for deviations were not described.
**Bias due to missing outcome data**	**Some concerns**	Attrition occurred in both groups (4 participants per arm), with reasons for withdrawal reported and similar across groups. However, the study did not specify whether an intention-to-treat analysis was conducted or how missing outcome data were handled, precluding a low-risk judgment.
**Bias in measurement of the outcome**	**Low risk**	Outcome assessors were reported to be unaware of group allocation, and standardized assessment instruments were used. Although complete blinding cannot be guaranteed in a cluster-based behavioral intervention, the methods described are sufficient to minimize risk of measurement bias.
**Bias in selection of the reported result**	**Some concerns**	Multiple outcomes and repeated assessments were analyzed, but no trial registration or prespecified statistical analysis plan was reported. As a result, selective reporting of outcomes or analyses cannot be excluded.

Overall risk of bias: High risk

Rationale: Despite the use of a cluster-randomized design, the inclusion of only two clusters with participant recruitment occurring after cluster allocation, together with marked baseline imbalances between intervention and control groups, resulted in a high risk of bias arising from the randomization process. This domain alone warrants an overall judgment of high risk of bias.

Bono 2015

RoB 2 domain	Judgment	Justification
**Bias arising from the randomization process**	**Some concerns**	The study is described as a randomized controlled trial; however, the methods do not provide sufficient detail on sequence generation or allocation concealment. Baseline characteristics appear broadly comparable between groups, but incomplete reporting precludes a low-risk judgment.
**Bias due to deviations from intended interventions**	**Some concerns**	Due to the nature of the animal-assisted intervention, blinding of participants and personnel was not feasible. Although the control group received follow-up at comparable intervals, the study does not report adherence monitoring or whether analyses accounted for deviations from the intended intervention.
**Bias due to missing outcome data**	**Some concerns**	Attrition occurred in both groups (4 participants per arm), resulting in analysis of 12 participants per group. Reasons for withdrawal were reported and attrition was balanced; however, the handling of missing data (e.g., intention-to-treat analysis or imputation) was not specified.
**Bias in measurement of the outcome**	**Some concerns**	Outcomes were assessed using standardized clinical scales; however, blinding of outcome assessors was not clearly described. Given the visible nature of the intervention, knowledge of group assignment may have influenced outcome assessment.
**Bias in selection of the reported result**	**Some concerns**	Multiple outcomes and time points were assessed, and no trial registration or prespecified analysis plan was reported. As such, selective reporting cannot be excluded.

Overall risk of bias: Some concerns

Rationale: Although the study employed a randomized controlled design with balanced attrition and reported reasons for withdrawal, insufficient methodological detail regarding randomization procedures, blinding, missing-data handling, and prespecification of analyses resulted in an overall judgment of some concerns for risk of bias.

Briones 2013

RoB 2 domain	Judgment	Justification
**Bias arising from the randomization process**	**Some concerns**	Participants meeting eligibility criteria were “randomly allocated by a ball draw” to intervention or control, but the report does not describe allocation concealment or implementation details (e.g., who performed the draw and whether assignment could be predicted). A notable baseline imbalance in functional status (Barthel Index) was present between groups , which raises concern about the integrity of the randomization process.

**Bias due to deviations from intended interventions**	**High risk**	Contamination is likely: all participants resided in the same facility and the dogs moved through the care home, allowing control participants to see and interact with the dogs; the authors explicitly state these interactions "were not reinforced, neither were they prevented," and that "everyone knew when the dogs came," implying nontrivial exposure among controls. This constitutes a deviation from intended intervention separation likely to bias the estimated between-group effect toward the null.
**Bias due to missing outcome data**	**High risk**	Outcome data were incomplete: the authors report inability to obtain measurements for some participants, including two deaths in the control group, and 7 missing QoL-AD assessments (2 intervention; 5 control). The handling of missing data (e.g., intention-to-treat analysis, imputation strategy) is not clearly specified, and missingness could plausibly be related to health status, introducing risk of bias.
**Bias in measurement of the outcome**	**Low risk**	Data collection instruments were administered by trained volunteers who were blind to experimental condition , supporting low risk of differential outcome measurement between groups.
**Bias in selection of the reported result**	**Some concerns**	Multiple outcomes and analyses were conducted (e.g., ANCOVA and mixed models) , but no trial registration or prespecified analysis plan is reported; therefore, selective reporting of outcomes or analyses cannot be excluded.

Overall RoB 2 judgment: High risk

Rationale: Although allocation was described as random, substantial risk of bias arises from contamination between groups within a shared facility and material missing outcome data without clearly described handling methods, which together warrant an overall judgment of high risk of bias.

Dabelko-Schoeny 2014

RoB 2 domain	Judgment	Justification
**Bias arising from the randomization process**	**Some concerns**	Participants were randomly assigned to the order of intervention and control periods using a computer-generated randomization list; however, participants were recruited from a single center using a convenience sample, and baseline functional and cognitive differences were observed between program groups, limiting confidence in the randomization process.
**Bias due to deviations from intended interventions**	**High risk**	Blinding of participants and staff was not feasible. The authors explicitly acknowledge that research assistants and staff were aware of intervention status, and intervention experiences may have varied by staff and group. These deviations from intended intervention conditions are likely to have influenced outcomes.
**Bias due to missing outcome data**	**Low risk**	No attrition or missing outcome data were reported. All participants completed both intervention and control periods and contributed repeated measures across time points.
**Bias in measurement of the outcome**	**High risk**	Behavioral outcomes were assessed using observational measures completed by staff and research assistants who were not blinded to intervention status, as acknowledged by the authors. This creates a high risk of differential outcome measurement in favor of the intervention.
**Bias in selection of the reported result**	**Some concerns**	Multiple behavioral and physiological outcomes were analyzed using several modeling approaches, and no trial registration or prespecified analysis plan was reported. Selective reporting of outcomes or analyses cannot be excluded.

Overall risk of bias: High risk

Rationale: Although the study employed a randomized crossover design with complete outcome data, the absence of blinding for participants, staff, and outcome assessors, together with acknowledged variability in intervention delivery, resulted in a high risk of bias due to deviations from intended interventions and outcome measurement.

Friedmann 2015

RoB 2 domain	Judgment	Justification
**Bias arising from the randomization process**	**Some concerns**	Facilities were paired and randomized by coin toss to intervention or control. Although baseline characteristics were broadly comparable between groups, the small number of clusters and limited reporting on allocation concealment preclude a low-risk judgment.
**Bias due to deviations from intended interventions**	**Low risk**	An attention-matched control intervention of equal frequency and duration was used, minimizing performance and attention bias. No concurrent activity programs were conducted during the study period, and analyses accounted for clustering and repeated measures.
**Bias due to missing outcome data**	**Low risk**	Attrition was limited (3/40 participants, all from the intervention group), reasons were reported, and formal testing indicated data were missing completely at random. Linear mixed-effects models appropriately handled incomplete longitudinal data.
**Bias in measurement of the outcome**	**Low risk**	Outcome assessments were conducted by an independent nurse observer not involved in the intervention. Validated and standardized instruments were used for all primary outcomes, reducing the likelihood of differential outcome assessment.
**Bias in selection of the reported result**	**Some concerns**	Multiple outcomes and time points were analyzed, and no trial registration or prespecified statistical analysis plan was reported. Selective reporting cannot be definitively excluded.

Overall risk of bias: Some concerns

Rationale: This cluster-randomized trial employed an attention-matched control, blinded outcome assessment, and appropriate longitudinal statistical methods. However, limited reporting of allocation concealment and absence of a prespecified analysis plan result in an overall judgment of *some concerns* for risk of bias.

Gregorini 2022

ROBINS-I domain	Judgment	Justification
**Bias due to confounding**	**Moderate risk**	Participants were not randomized to intervention; however, groups were deliberately balanced by diagnosis (AD vs non-AD), group size (n=6 per subgroup), sex distribution, and residence within the same facility. Baseline comparisons showed general homogeneity across clinical and neuropsychological parameters, with the exception of a higher baseline diastolic blood pressure in the AD-AAT group. Although no multivariable adjustment was performed, the small, structured design and baseline reporting reduce the risk of confounding.
**Bias in selection of participants into the study**	**Moderate risk**	Participants were selected from a single rest house using predefined inclusion and exclusion criteria (e.g., ICD-10 diagnosis, exclusion of aggressive behavior, immunodepression, dog allergy). The method of assignment to AAT versus control groups was not described in detail, raising the possibility that selection was influenced by prognosis or feasibility rather than chance.
**Bias in classification of interventions**	**Low risk**	Intervention status was clearly defined. AAT consisted of standardized weekly sessions over 10 weeks, and control participants were explicitly prevented from seeing or interacting with animals, minimizing misclassification.
**Bias due to deviations from intended interventions**	**Moderate risk**	Blinding of participants and staff was not feasible. Although sessions were protocolized and residents maintained usual daily routines, additional attention or engagement related to AAT outside scheduled sessions cannot be excluded and was not formally assessed.
**Bias due to missing data**	**Low risk**	The study does not report attrition or missing outcome data across intervention sessions or follow-up assessments. Outcomes are consistently presented at all planned time points, suggesting minimal impact of missing data.
**Bias in measurement of outcomes**	**Low risk**	Objective physiological measures (blood pressure and heart rate) were used. Neuropsychological outcomes (MMSE and GDS) were administered by a psychologist who was explicitly blinded to group assignment, reducing the likelihood of differential outcome measurement.
**Bias in selection of the reported result**	**Moderate risk**	Multiple outcomes and repeated analyses were conducted, and no prespecified protocol or trial registration was reported. While comprehensive reporting is provided, selective reporting of analyses cannot be definitively excluded.

Overall ROBINS-I judgment: Moderate

Rationale: Although the study was non-randomized, participant groups were deliberately balanced by diagnosis and size, baseline characteristics were largely comparable, and outcome assessment incorporated blinded neuropsychological evaluation and objective physiological measures. Nevertheless, incomplete reporting of allocation procedures and lack of adjustment for residual baseline differences result in an overall judgment of moderate risk of bias.

Kanamori 2001

ROBINS-I domain	Judgment	**Jus**tification
**Bias due to confounding**	**Serious risk**	Allocation to AAT was based on participant preferences and prior experience: the AAT group consisted of individuals who liked animals and had experience keeping them. This introduces strong risk of confounding by baseline psychosocial traits (e.g., affinity to animals, engagement) that are plausibly related to behavioral outcomes (Behave-AD), independent of the intervention effect. In addition, dementia subtype distribution differed between groups (DAT/VD ratios not matched) , which could further confound symptom trajectories.
**Bias in selection of participants into the study**	**Serious risk**	Participants were selected into the intervention group explicitly based on liking animals and prior animal experience , creating a selection process that is very likely related to prognosis/response (especially for affective/behavioral endpoints).
**Bias in classification of interventions**	**Low risk**	Intervention status is clearly defined (AAT delivered as six biweekly sessions over ~3 months), and controls explicitly did not receive AAT.
**Bias due to deviations from intended interventions**	**Moderate risk**	Blinding was not described and is unlikely in a day care intervention involving animals. Co-interventions related to day care activities may have contributed to behavioral changes; authors explicitly note difficulty attributing improvements to AAT versus day care participation.
**Bias due to missing data**	**Moderate risk**	For primary scales (MMSE/N-ADL/Behave-AD), missingness is not reported and summary results are presented for the full stated n. However, important secondary outcome sampling was incomplete (CgA: 4 AAT; 14 control), suggesting nonrandom missingness for stress outcomes.

**Bias in measurement of outcomes**	**Moderate risk**	Behavioral outcomes (Behave-AD) were obtained via interviews with family, with no indication of blinded assessment; this increases risk of measurement bias, particularly when caregivers know the participant received AAT. Cognitive/ADL scales are standardized, but assessor blinding is not described.
**Bias in selection of the reported result**	**Moderate risk**	Multiple outcomes were assessed (MMSE, N-ADL, Behave-AD subscales, salivary CgA), and no protocol/registration is reported. Selective reporting cannot be excluded.

Overall ROBINS-I judgment: Serious risk of bias

Rationale: As intervention allocation was explicitly based on liking animals and prior animal experience, substantial confounding and selection bias are likely for behavioral outcomes, warranting an overall rating of serious risk of bias despite clear intervention classification and use of standardized scales.

Kruger 2022

ROBINS-I domain	Judgment	Justification
**Bias due to confounding**	**Low risk**	Each participant served as their own control, substantially reducing confounding by stable patient-level characteristics (e.g., dementia severity, comorbidities, baseline agitation). Medication use, coercive measures, and contact time were documented on both days, and no systematic differences were observed between intervention and control days that would plausibly explain the observed effect.
**Bias in selection of participants into the study**	**Moderate risk**	Participants were recruited from a single inpatient ward and inclusion required the presence of agitation and consent by legal guardians. While selection may limit generalizability, there is no evidence that inclusion was related to differential response between intervention and control conditions within individuals.
**Bias in classification of interventions**	**Low risk**	Intervention status (dog present vs absent) was clearly defined, contemporaneous, and precisely documented. Misclassification of exposure is unlikely.
**Bias due to deviations from intended interventions**	**Moderate risk**	The sequence of intervention and control days was not randomized, and the dog handler was also the ward physician, raising the possibility of subtle behavioral differences across days. However, contact time was measured and did not differ significantly between conditions, mitigating concern for systematic deviation.
**Bias due to missing data**	**Moderate risk**	Attrition prior to analysis occurred due to clinical deterioration and discharge. Physiological outcomes (HRV, BDNF) had substantial missingness, but the primary behavioral outcome (OASS) was available for all analyzed participants, limiting impact on the main effect estimate.
**Bias in measurement of outcomes**	**Low risk**	Agitation was assessed using the OASS by a trained rater blinded to the presence of the dog, with standardized observation periods. This substantially reduces the risk of differential outcome assessment.
**Bias in selection of the reported result**	**Low risk**	The trial was prospectively registered (DRKS00024093), and outcomes reported align with those described in the methods, reducing concern for selective reporting.

Overall risk of bias: Moderate

Rationale: This investigator-blinded within-subject study minimizes confounding through self-control and employs standardized, blinded outcome assessment. Residual concerns relate primarily to nonrandomized sequencing and missing physiological data, but these limitations do not materially compromise the internal validity of the primary agitation outcome.

Majic 2013

RoB 2 domain	Judgment	Justification (table-ready wording)
**Bias arising from the randomization process**	**Some concerns**	The paper states that matched pairs were randomly assigned to TAU vs TAU + AAT ; however, the method of sequence generation and allocation concealment is not described. In addition, AAT was only implemented in 2 nursing homes with controls drawn from 6 homes , increasing the risk that contextual/site factors affected group comparability despite matching.
**Bias due to deviations from intended interventions**	**Some concerns**	Blinding of participants and staff was not feasible. Both groups received TAU, and the intervention consisted of TAU plus structured AAT sessions. Co-interventions may have differed by facility (AAT homes vs non-AAT homes), but deviations were not systematically reported.
**Bias due to missing outcome data**	**Some concerns**	Ten participants dropped out prior to final analytic sample formation (5 per group), and an additional 11 were excluded because they could not be matched. Although the matched sample (n=54) appears complete for analysis, exclusions related to matching may introduce bias if excluded participants differed systematically.
**Bias in measurement of the outcome**	**Some concerns**	Outcomes were derived from interviews with patients and professional caregivers conducted by trained raters. The report does not clearly state that outcome assessors were blinded to allocation, and caregiver-informed measures (CMAI/DMAS) are susceptible to detection bias when group assignment is known.

**Bias in selection of the reported result**	**Some concerns**	Analyses used repeated-measures ANCOVA with covariates and matched-pair denominators. No trial registration or prespecified analysis plan is reported in the paper; selective reporting cannot be excluded.

Overall risk of bias: Some concerns

Rationale: Although the study is described as randomized within a matched-pair framework, limited reporting of allocation procedures, substantial exclusions due to matching, unclear assessor blinding, and the restriction of AAT delivery to a subset of nursing homes support an overall judgment of some concerns for risk of bias.

Menna 2016

ROBINS-I domain	Judgment	Justification
**Bias due to confounding**	**Moderate risk**	Group allocation was non-randomized and partially based on willingness and prior familiarity with animals. Although baseline age, sex, MMSE, and GDS were similar across groups, unmeasured psychosocial factors (e.g., motivation, affinity toward animals) may have influenced outcomes.
**Bias in selection of participants**	**Moderate risk**	Participants were selected from a single center and allocated sequentially rather than randomly. Selection procedures were transparent but may limit internal validity and generalizability.
**Bias in classification of interventions**	**Low risk**	Intervention conditions (AAT + ROT, ROT only, no stimulation) were clearly defined, structured, and consistently implemented over 6 months.
**Bias due to deviations from intended interventions**	**Moderate risk**	Participants and providers were not blinded, and the zootherapist played an active role in AAT delivery. However, intervention fidelity was standardized and comparable contact time was maintained across active groups.
**Bias due to missing data**	**Low risk**	Outcome data were complete for all participants, with no reported attrition or differential loss to follow-up.
**Bias in measurement of outcomes**	**Moderate risk**	MMSE and GDS were administered by clinicians aware of group assignment, introducing potential detection bias, particularly for mood outcomes. Objective scoring partially mitigates this concern.
**Bias in selection of reported results**	**Low risk**	Outcomes specified in methods were fully reported, with consistent analyses across groups and time points.

Overall risk of bias: Moderate

Rationale: This pilot controlled study demonstrates careful baseline comparability, complete outcome reporting, and standardized intervention delivery. However, non-randomized allocation, lack of blinding, and potential residual confounding limit causal inference, resulting in an overall moderate risk of bias.

Moretti 2011

ROBINS-I domain	Judgment	Justification
**Bias due to confounding**	**Moderate risk**	Allocation was non-randomized; however, cases and controls were deliberately selected to be similar in sociodemographic and clinical characteristics (age, sex, education, diagnosis distribution, baseline MMSE and GDS). Although unmeasured psychosocial factors (e.g., motivation, affinity toward animals) may still confound results, no major baseline imbalances were observed.
**Bias in selection of participants into the study**	**Moderate risk**	Participants were selected from a single nursing home and assigned to groups based on matching rather than random allocation. Selection procedures were transparent but may limit internal validity and generalizability.
**Bias in classification of interventions**	**Low risk**	Exposure to pet therapy was clearly defined, structured, and consistently delivered; control participants did not receive structured animal interaction.
**Bias due to deviations from intended interventions**	**Moderate risk**	Blinding of participants and staff was not feasible. Control participants were allowed to see animals entering the facility, and informal contact was not prohibited, raising the possibility of contamination.
**Bias due to missing data**	**Low risk**	No attrition or missing outcome data were reported after baseline; all enrolled participants completed follow-up assessments.
**Bias in measurement of outcomes**	**Low risk**	MMSE and GDS were administered by a trained physician **blinded to group allocation**, substantially reducing risk of detection bias.
**Bias in selection of the reported result**	**Moderate risk**	Multiple outcomes were assessed and no protocol or registration was reported; selective reporting cannot be excluded, although outcomes described in the methods were reported in full.

Overall risk of bias: Moderate

Rationale: Although the study was non-randomized, careful matching of cases and controls, blinded outcome assessment, and complete follow-up mitigate major threats to internal validity. Residual confounding and potential contamination support an overall classification of moderate risk of bias.

Moyle 2018

RoB 2 domain	Judgment	Justification
**Bias arising from the randomization process**	**Low risk**	Facilities were randomised by an independent, centralised, computer-generated sequence, stratified by facility type and allocated in blocks. Allocation was concealed from facilities, staff, participants, and families until intervention commencement. Baseline characteristics were well balanced across groups.
**Bias due to deviations from intended interventions**	**Low risk**	The intervention was delivered according to a prespecified protocol. Masking of participants was not feasible, but research assistants were assigned to a single study arm, reducing contamination. Analyses followed an intention-to-treat framework.
**Bias due to missing outcome data**	**Some concerns**	A substantial proportion of participants were excluded from analyses due to insufficient accelerometer wear-time. Although missingness was related to tolerability rather than outcomes, last-observation-carried-forward was used, which may introduce bias if missingness differed by outcome trajectory.
**Bias in measurement of the outcome**	**Low risk**	Outcomes were objectively measured using wearable triaxial accelerometers. Research assistants involved in data collection and coding were masked through assignment to a single intervention group, reducing detection bias.
**Bias in selection of the reported result**	**Low risk**	The trial was prospectively registered, and reported outcomes and analyses were consistent with the prespecified protocol. Mixed-effects models appropriately accounted for clustering.

Overall risk of bias: Some concerns

Rationale: This well-conducted cluster-randomized trial employed robust allocation procedures, objective outcome measurement, and intention-to-treat analyses. However, substantial missing accelerometer data and reliance on last-observation-carried-forward imputation introduce some uncertainty, supporting an overall judgment of some concerns.

Olsen 2016a

RoB 2 domain	Judgment	Justification
**Bias arising from the randomization process**	**Some concerns**	Clusters (nursing home units) were randomized using computer-generated random numbers , but allocation concealment and baseline balance at the cluster level are not described in sufficient detail, and participant recruitment was performed by nursing home staff within clusters, which can introduce selection bias in cluster trials.
**Bias due to deviations from intended interventions**	**Some concerns**	Treatment-as-usual control and AAA were delivered as planned and facilities were required to abstain from other dog-visiting activities during the study period ; however, blinding of participants/staff is inherently difficult and differential co-interventions/expectancy effects cannot be ruled out in this setting.
**Bias due to missing outcome data**	**Some concerns**	Attrition occurred including deaths and the authors report a ~17% dropout rate. While attrition is plausible in this population, the potential for missingness related to outcomes remains and imputation was used for item-level missingness .
**Bias in measurement of the outcome**	**High risk**	Outcomes were rated by nursing home personnel and raters were not blinded to group assignment. Depression (CSDD), agitation (BARS), and QoL (QUALID) are rater-dependent measures, so lack of blinding could plausibly influence scoring.
**Bias in selection of the reported result**	**Low risk**	Trial registration is reported (ClinicalTrials.gov NCT02008630) and the main outcomes specified (depression, agitation, QoL) are consistently presented .

Overall risk of bias: High risk

Rationale: High risk in outcome measurement due to non-blinded raters.

Olsen 2016b

RoB 2 domain	Judgment	Justification
**Bias arising from the randomization process**	**Some concerns**	Day-care centers were randomized using computerized random numbers before participant recruitment. However, randomization occurred at the cluster level prior to individual enrollment, and participants were subsequently recruited by staff within centers, creating potential for recruitment-related selection bias. Baseline imbalance was observed in BBS scores, with the intervention group scoring significantly lower at pre-test.
**Bias due to deviations from intended interventions**	**Some concerns**	Blinding of participants and staff was not feasible. The intervention was delivered according to a standardized protocol and centers abstained from dog activities outside the intervention. Nonetheless, expectancy and performance effects cannot be excluded in a non-blinded behavioral intervention.

**Bias due to missing outcome data**	**Some concerns**	Attrition was low (one withdrawal), but missing outcome data occurred at item and scale level. The authors used multiple imputation and person-mean substitution, which reduces but does not eliminate the risk that missingness was related to outcome or group assignment.
**Bias in measurement of the outcome**	**High risk**	Outcome assessors were not blinded to group allocation. Both primary outcomes (BBS and QUALID) involve rater judgment, and the authors explicitly acknowledge that non-blinded assessment may have influenced results, particularly for BBS.
**Bias in selection of the reported result**	**Low risk**	The trial was prospectively registered (ClinicalTrials.gov NCT02008630), and prespecified primary outcomes and analytic methods (mixed models accounting for clustering) were reported consistently.

Overall risk of bias: High risk

Rationale: Although this cluster-randomized trial was prospectively registered and used appropriate statistical models accounting for clustering and repeated measures, the lack of blinding of outcome assessors for rater-dependent primary outcomes (balance and proxy-rated quality of life), combined with baseline imbalance and potential recruitment bias inherent to cluster randomization prior to individual enrollment, represents a serious threat to internal validity.

Petersen 2017

RoB 2 domain	Judgment	Justification
**Bias arising from the randomization process**	**Some concerns**	Participants were randomly assigned to intervention or control using a coin-toss method until sample size was achieved. Although baseline characteristics were reported as comparable between groups (Table 1), the method of sequence generation and allocation concealment lacks rigor and transparency, and block structure is not fully described.
**Bias due to deviations from intended interventions**	**Some concerns**	Blinding of participants and nursing staff was not feasible. Both groups received structured programming, and the intervention added PARO exposure; however, the principal investigator and trained facility nurses delivered sessions and also collected outcome data, increasing the possibility of performance effects despite standardized protocols.
**Bias due to missing outcome data**	**Some concerns**	Longitudinal physiologic measures were collected repeatedly across sessions, but the extent and handling of missing repeated-measure data are not fully specified. Attrition and incomplete session data are plausible in this population and may be related to health status.
**Bias in measurement of the outcome**	**High risk**	Primary outcomes were staff-rated or clinician-rated, and assessors were not blinded to group assignment. Although physiologic measures are objective, key behavioral outcomes are susceptible to detection bias in a non-blinded context.
**Bias in selection of the reported result**	**Some concerns**	Multiple outcomes and analyses were conducted, including t-tests, GLM, and mixed models. No prospective trial registration or prespecified statistical analysis plan is reported, so selective reporting of outcomes or analytic approaches cannot be excluded.

Overall risk of bias: High risk

Rationale: Although this randomized controlled trial demonstrated baseline group comparability and incorporated both behavioral and objective physiological outcomes, the lack of blinding for rater-dependent primary outcomes, coupled with limited reporting on allocation concealment and incomplete specification of missing longitudinal data handling, represents a substantial threat to internal validity.

Quintavalla 2021

RoB 2 domain	Judgment	Justification
Bias arising from the randomization process	**Some concerns**	The authors state that participants were “randomly selected” and that both intervention and control groups were randomly constituted. However, the method of sequence generation, allocation concealment, and implementation of randomization are not described, and the resulting group sizes were markedly imbalanced (30 vs 10), limiting confidence that randomization fully prevented selection bias.
Bias due to deviations from intended interventions	**Some concerns**	Blinding of participants and staff was not feasible in a dog-assisted intervention. Both groups continued standard day-care activities, but the intervention group received additional structured interaction and attention, and no attention-matched control was used, allowing potential performance effects.
Bias due to missing outcome data	**Some concerns**	Mortality occurred during the study (3 intervention, 1 control). The paper does not clearly state whether analyses were intention-to-treat or how missing data from deceased participants were handled, raising concern that missingness related to health status could influence estimates.
Bias in measurement of the outcome	**Some concerns**	Outcomes included standardized cognitive scales (MMSE, ADAS) and a perceived wellbeing questionnaire (BAC). The study does not report blinding of outcome assessors, and for rater-administered or proxy-reported measures, awareness of group assignment could plausibly influence scoring.
Bias in selection of the reported result	**Some concerns**	Multiple outcomes and repeated time-point analyses were conducted, but no trial registration or prespecified analysis plan is reported. Selective reporting of outcomes or analytic contrasts cannot be excluded.

Overall risk of bias: Some concerns

Rationale: Although Quintavalla et al. describe random constitution of intervention and control groups, insufficient reporting of the randomization procedure, pronounced group-size imbalance, lack of assessor blinding for rater-dependent outcomes, and unclear handling of missing data due to mortality introduce uncertainty regarding internal validity.

Robinson 2013

RoB 2 domain	Judgment	Justification
**Bias arising from the randomization process**	**Low risk**	Participants were randomized using a random list generator, with equal allocation to Paro and control groups. Baseline characteristics, including cognitive impairment, were comparable between groups, indicating successful randomization.
**Bias due to deviations from intended interventions**	**Some concerns**	Blinding of participants and staff was not feasible. The researcher was present during some Paro sessions to facilitate activities, which may have enhanced engagement. However, the researcher was also present during control activities, reducing differential performance bias.
**Bias due to missing outcome data**	**Some concerns**	Attrition occurred equally in both groups (3/20 each), primarily due to death. Analyses were conducted on complete cases only, and no intention-to-treat analysis or imputation strategy was reported, raising concern that missingness related to health decline could affect estimates.
**Bias in measurement of the outcome**	**Some concerns**	Outcomes included validated self-report and proxy-rated instruments (UCLA Loneliness Scale, GDS, QoL-AD). The paper does not state that outcome assessors were blinded to group allocation; for subjective and proxy-rated outcomes, awareness of intervention status could influence scoring.
**Bias in selection of the reported result**	**Low risk**	The trial was prospectively registered (ANZCTR 12612000469819), and all primary outcomes described in the methods were reported using prespecified ANCOVA analyses adjusting for baseline scores.

Overall risk of bias: Some concerns

Rationale: This individually randomized controlled trial used an appropriate randomization procedure, demonstrated baseline group comparability, and was prospectively registered with prespecified outcomes. However, unavoidable lack of blinding for subjective psychosocial outcomes and complete-case analysis in the presence of attrition due to mortality introduce residual uncertainty regarding effect estimates.

Rodrigo-Claverol 2020

ROBINS-I domain	Judgment	Justification
**Bias due to confounding**	**Serious risk**	Allocation to intervention was by nursing home center (Center 1 = EG; Center 2 = CG) because dog presence could not be masked, creating strong potential for center-level confounding (environment, staffing, culture). Although baseline demographic and GDS severity distributions were broadly similar, baseline imbalance existed for Tinetti balance (EG worse; p=0.041) , and baseline adjustment was performed in regression models ; however, unmeasured cluster-level factors remain plausible and cannot be fully controlled statistically.
**Bias in selection of participants into the study**	**Moderate risk**	Participants were recruited from two centers with predefined inclusion/exclusion criteria, but group membership was determined by center assignment. For equity, a subset of eligible residents in the larger control center was randomly invited , which reduces but does not eliminate potential selection differences between centers and limits generalizability.
**Bias in classification of interventions**	**Low risk**	Intervention status was clearly defined (same structured program with vs without therapy dog), and misclassification is unlikely given center-based delivery and standardized session structure.
**Bias due to deviations from intended interventions**	**Moderate risk**	The trial was open-label and blinding was not feasible. Separating centers reduced contamination, and the same professionals delivered sessions in both groups (apart from the dog), which mitigates differential co-interventions; nevertheless, expectancy and performance effects are plausible in animal-assisted interventions.
**Bias due to missing data**	**Moderate risk**	Dropout was 8% in each group with final balanced sample sizes , which limits differential attrition. However, reasons for dropout are not detailed, and in frail institutionalized populations missingness can be related to health decline, potentially affecting outcome estimates.
**Bias in measurement of outcomes**	**Moderate risk**	Outcomes were measured with standardized scales (Holden, Tinetti) , but assessor blinding is not reported and the design is explicitly open-label. For rater-scored outcomes, knowledge of intervention status could influence scoring, especially for communication.
**Bias in selection of the reported result**	**Moderate risk**	Multiple outcomes and subgroup analyses (by GDS) were presented. No prospective trial registration or prespecified analysis plan is reported in the manuscript, so selective reporting of analyses cannot be excluded, although main outcomes are consistently described and analyzed using regression controlling for baseline scores.

Overall risk of bias: Serious

Rationale: This controlled nonrandomized cluster trial used a structured, standardized intervention delivered by the same professionals in both arms and applied validated outcome measures for communication and mobility. However, because allocation was determined by nursing home

center (to avoid contamination from visible dog exposure), the comparison is vulnerable to residual center-level confounding despite baseline comparability checks and statistical adjustment for baseline scores. The open-label nature of the intervention and unclear blinding of outcome assessors further increase susceptibility to bias for rater-scored outcomes.

Schuurmans 2021

RoB 2 domain	Judgment	Justification
**Bias arising from the randomization process**	**Some concerns**	Allocation to intervention arms was randomized at the cluster level using computer-generated numbers in one large location and cluster-level assignment in smaller locations . However, mixed randomization strategies (individual vs cluster), small cluster sizes, and baseline age imbalance (younger-onset dementia cluster randomized to dog group) introduce uncertainty regarding baseline equivalence across groups.
**Bias due to deviations from intended interventions**	**Some concerns**	Blinding of participants and staff was not feasible. Sessions followed a standardized protocol across groups, and handler initiative was explicitly measured and shown not to differ between groups, which mitigates but does not eliminate performance bias.
**Bias due to missing outcome data**	**High risk**	There was substantial and differential loss to follow-up, particularly in the control group where nearly all participants from one location discontinued after the first session. Subsequent analyses excluded participants with <4 session attendance, resulting in a markedly reduced and selective analytic sample. Missingness is plausibly related to intervention acceptability and outcomes.
**Bias in measurement of the outcome**	**Low risk**	Outcomes were derived from structured video recordings coded by trained observers using a predefined ethogram. Inter-rater reliability was high (Cohen's $\kappa \approx 0.7–0.9$). Observers were not involved in intervention delivery, reducing detection bias for behavioral outcomes.
**Bias in selection of the reported result**	**Some concerns**	The study was prospectively registered (ISRCTN93568533), but due to extensive attrition, between-group comparisons were limited and alternative analytic strategies (e.g., bootstrapped one-sample tests vs zero) were used. Selective emphasis on within-group findings cannot be fully excluded.

Overall risk of bias: High risk

Rationale: Although this cluster-randomized trial employed a standardized intervention protocol, prospective registration, and rigorous video-based behavioral coding with high inter-rater reliability, the internal validity is substantially compromised by severe and differential attrition across intervention arms. Exclusion of participants with low attendance and near-complete loss of one control cluster introduce a high likelihood that analyzed outcomes are not representative of the randomized population.

Travers 2013

RoB 2 domain	Judgment	Justification
**Bias arising from the randomization process**	**Some concerns**	Participants were randomly assigned within each facility using a simple procedure ("names drawn from a hat"). This is a random mechanism, but allocation concealment and safeguards against predictability are not described, leaving uncertainty about whether assignment could have been anticipated or influenced.
**Bias due to deviations from intended interventions**	**Low risk**	Both groups received highly standardized, semi-structured therapist-led sessions delivered by the same therapist, with the dog being the key difference between arms. Contamination was minimized by preventing control participants from having any dog contact (dog housed in a covered crate in a secure adjacent area during control sessions).
**Bias due to missing outcome data**	**Some concerns**	Attrition was ~18% overall (67 randomized; 55 completers). Reasons included death, hospitalization, family withdrawal, and pre-specified exclusion for repeated non-attendance. Losses were broadly similar across groups, but analyses were based on completers and no intention-to-treat approach or imputation strategy was reported.
**Bias in measurement of the outcome**	**Some concerns**	The study psychologist administering participant-reported outcomes was blinded to group allocation (reducing detection bias for QOL-AD, SF-36, and GDS). However, facility staff who completed the MOSES were explicitly not blinded, and MOSES subscales are observer-rated and susceptible to expectancy effects.
**Bias in selection of the reported result**	**Some concerns**	Multiple outcomes and interaction analyses (facility effects and baseline-score interactions) were undertaken. No prespecified statistical analysis plan or prospective trial registration is reported, so selective emphasis on significant contrasts cannot be fully excluded, although outcomes described in the methods were presented in the results tables.

Overall risk of bias: Some concerns

Rationale: This multicenter randomized trial used structured, attention-matched control sessions delivered by the same therapist and implemented strong contamination control procedures. Blinded outcome administration for participant-reported measures is a major strength. However, incomplete reporting of allocation concealment, reliance on complete-case analyses in the presence of attrition, and non-blinded staff-rated behavioral assessments (MOSES) introduce residual uncertainty regarding internal validity.

Valenti-Soler 2015

RoB 2 domain	Judgment	Justification
**Bias arising from the randomization process**	**Some concerns**	Randomization was performed by living units (clusters) stratified by dementia severity, using a six-sided die before baseline assessment . Baseline comparability was demonstrated for most outcomes, but cluster-level allocation and occasional baseline imbalance (e.g., NPI irritability in Phase 2) limit certainty that randomization fully eliminated selection bias.
**Bias due to deviations from intended interventions**	**Low risk**	Interventions were delivered by the same therapists, with identical session structure, timing, and environment across groups. Robots/dogs were introduced strictly as therapeutic tools without altering content. Separation by living units minimized contamination.
**Bias due to missing outcome data**	**Low risk**	Attrition during intervention phases was minimal (<3%), and most losses occurred outside intervention windows. Missingness was unlikely to be related to intervention assignment or outcomes.
**Bias in measurement of the outcome**	**Low risk**	Outcomes were assessed by blinded raters (neurologist, neuropsychologist, psychiatrist, sociologist), using validated scales. Video acclimatization was used to reduce Hawthorne effects.
**Bias in selection of the reported result**	**Some concerns**	Multiple outcomes and item-level analyses were conducted without correction for multiple comparisons, and no public trial registration or prespecified statistical analysis plan is reported. Selective emphasis on significant findings cannot be fully excluded.

Overall risk of bias: Some concerns

Rationale: Across both phases, this cluster-randomized trial was conducted in a highly controlled institutional setting with standardized therapy delivery, blinded outcome assessment, and very low attrition, representing notable methodological strengths. However, cluster-level randomization by living unit, occasional baseline imbalances, and extensive outcome testing without a prespecified analytic hierarchy introduce residual uncertainty regarding internal validity.

Vegue-Parra 2021

RoB 2 domain	Judgment	Justification
**Bias arising from the randomization process**	**Low risk**	Participants were individually randomized to intervention or control groups after eligibility screening, with stratification by cognitive deterioration, age, sex, and other variables to ensure group homogeneity. Baseline comparisons showed no significant differences between groups across outcome measures, supporting successful randomization.
**Bias due to deviations from intended interventions**	**Some concerns**	Blinding of participants and care staff was not feasible due to the nature of dog-assisted therapy. However, both groups continued to receive standard center-based therapies, and no evidence of systematic co-intervention imbalance is reported. Performance effects related to attention and novelty cannot be fully excluded.
**Bias due to missing outcome data**	**Some concerns**	Attrition occurred in both groups (≈10%), mainly due to death, transfer, or abandonment. Analyses appear to be based on complete cases, and no explicit intention-to-treat analysis or imputation strategy is described, leaving uncertainty regarding the impact of missingness related to health status.
**Bias in measurement of the outcome**	**Low risk**	Outcome assessments were conducted by occupational therapists or psychologists blinded to group allocation, and a double-blind procedure is explicitly described for data collection and analysis. Validated instruments were used for all outcome domains, reducing risk of detection bias.
**Bias in selection of the reported result**	**Some concerns**	Multiple outcomes and subgroup analyses were conducted across three time points. No prospective trial registration or prespecified statistical analysis plan is reported, and multiple testing was performed without correction, so selective emphasis on significant findings cannot be excluded.

Overall risk of bias: Some concerns

Rationale: This large multicenter randomized controlled trial demonstrates several methodological strengths, including individual randomization with stratification, baseline group comparability, long intervention duration, and blinded outcome assessment using validated instruments. Nonetheless, unavoidable lack of participant blinding, reliance on complete-case analyses in the presence of attrition, and absence of a prespecified analysis plan introduce residual uncertainty regarding effect estimates.

Wesenberg 2019

ROBINS-I domain	Judgment	Justification
**Bias due to confounding**	**Moderate**	The within-subject design reduces stable between-person confounding because participants serve as their own control . However, residual time-varying confounding remains plausible (disease progression, fluctuating symptoms, medication changes were documented but not clearly adjusted for) . In addition, carryover effects are explicitly acknowledged as a limitation in this design .
**Bias in selection of participants into the study**	**Moderate**	Participants were a small feasibility sample from two nursing homes with inclusion/exclusion criteria (e.g., language, stable dementia meds, no contraindications such as allergy/phobia/immobility), which may select a more tolerant/healthier subgroup and limit representativeness .
**Bias in classification of interventions**	**Low**	Intervention status (dog present vs no dog) is unambiguous, prespecified, and delivered as distinct manualized weekly sessions for 6 months . Misclassification is very unlikely.
**Bias due to deviations from intended interventions**	**Moderate**	Blinding is not feasible (dog presence is obvious). Group facilitators differ (dog-owner teams vs volunteers), and the paper notes this as a potential limitation even though training and demographic matching were attempted . Contamination/carryover is possible despite scheduling sessions on separate weekdays .
**Bias due to missing data**	**Moderate**	There were 2 early dropouts (death; withdrawal without reason). Analyses are essentially on completers (n=17), and with such a small sample, even limited attrition could meaningfully influence results; no imputation/ITT strategy is described.
**Bias in measurement of outcomes**	**Serious**	Outcomes were derived from video-coded behaviors and emotions. While inter-rater reliability procedures are described ($\kappa > 0.6$) , blinding of raters to condition is not feasible because the dog is visible. This creates a meaningful risk of detection/observer bias for subjective behavioral and affective coding.
**Bias in selection of the reported result**	**Moderate**	Multiple outcomes and repeated time points were analyzed with several statistical comparisons (Wilcoxon tests, Friedman tests; FDR adjustment). No protocol/registration is provided here, so selective emphasis of outcomes cannot be excluded.

Overall risk of bias: Serious

Rationale: Although the within-subject design helps reduce baseline confounding, this small feasibility study remains vulnerable to carryover/time effects, complete-case analysis after attrition, and serious risk of outcome measurement bias because video raters cannot be blinded to dog presence for subjective behavioral/emotional outcomes.

William 2016

RoB 2 domain	Judgment	Justification
**Bias arising from the randomization process**	**Some concerns**	Participants were assigned in random order to AAT or human interaction blocks . However, the method of sequence generation and allocation concealment is not described in detail, limiting certainty about protection against selection bias. Baseline equivalence is inherently controlled in a crossover design.
**Bias due to deviations from intended interventions**	**Low risk**	Both conditions were delivered according to standardized protocols, matched for time and attention. A one-week washout period was implemented between conditions, and sequence-effect testing showed no significant carryover effects, supporting adherence to intended interventions.
**Bias due to missing outcome data**	**Low risk**	No attrition or missing outcome data were reported during the intervention phases. All participants contributed data to both treatment conditions.
**Bias in measurement of the outcome**	**Some concerns**	Outcomes relied on direct observation and staff-informed ratings (social behavior checklist, MPES, CMAI). Although extensive observer training and high inter-rater reliability (>80–90%) were reported, blinding of observers was not feasible because dog presence was obvious, introducing potential detection bias for subjective measures.
**Bias in selection of the reported result**	**Some concerns**	Multiple outcomes and time windows (pre, during, post visits) were analyzed using ANOVA and nonparametric tests. No prospective trial registration or prespecified statistical analysis plan is reported, and multiplicity adjustment was not described.

Overall risk of bias: Some concerns

Rationale: This randomized crossover trial benefits from strong internal control through within-participant comparisons, implementation of a washout period, and absence of attrition. Nevertheless, incomplete reporting of the randomization procedure and unavoidable lack of blinding for observer-rated behavioral outcomes introduce residual uncertainty.
