# Supplementary material for: Animal-Assisted Therapy in Dementia: A Systematic Review and Meta-Analysis of Cognitive, Affective, and Behavioral Outcomes
Source: J Clin Med. 2026 Aug 28;15(17):6654. doi: 10.3390/jcm15176654 (PMC13566663; doi:10.3390/jcm15176654)
Supplement: Supplementary file 1 [file jcm-15-06654-s001.zip › Supplementary_Table_S5.pdf]

Supplementary Table S5. GRADE Summary of Findings

Outcome	Studies (comparisons)	Participants	Effect (SMD, 95% CI)	Certainty	Reasons for downgrading
Overall BPSD severity	5 (6)	616	−0.53 (−0.91 to −0.15)	⨁○○○ Very low	Risk of bias (−1): no study at low risk; two randomized trials at high risk [11,14] and one non-randomized study at serious risk [28]. Inconsistency (−1): I^2 = 73%. Imprecision (−1): effect lost significance on exclusion of high-risk studies; six comparisons only.
Depressive symptoms	11 (11)	684	−0.79 (−1.33 to −0.26)	⨁⨁○○ Low	Risk of bias (−1): no study at low risk; two randomized trials at high risk [11,18]; three non-randomized studies included [27,30,31]. Inconsistency (−1): I^2 = 87%. Not downgraded for imprecision: the confidence interval excluded no effect in both the primary analysis and the analysis restricted to randomized trials (−0.58, −1.17 to −0.01), and the optimal information size was met (684 participants).
Cognitive performance	9 (9)	617	1.17 (0.31 to 1.79)	⨁○○○ Very low	Risk of bias (−1): no study at low risk; one randomized trial at high risk [11]; four non-randomized studies including one at serious risk [28]. Inconsistency (−1): I^2 = 92%; two studies [21,30] contribute effect sizes above g = 3. Imprecision (−1): wide interval; effect not robust to exclusion of outlying studies.
Agitation	5 (5)	256	−0.81 (−1.68 to 0.06)	⨁○○○ Very low	Risk of bias (−1): no study at low risk; one randomized trial at high risk [18]. Inconsistency (−1): I^2 = 90%. Imprecision (−1): interval includes no effect; 256 participants across five comparisons.
Quality of life	6 (8)	326	0.12 (−0.26 to 0.50)	⨁⨁○○ Low	Risk of bias (−1): all comparisons from randomized trials, but three at high risk [13,18,19] and none at low risk. Imprecision (−1): interval includes no effect; 326 participants. Not downgraded for inconsistency (I^2 = 60%).
Activities of daily living	5 (5)	447	0.66 (−0.18 to 1.50)	⨁○○○ Very low	Risk of bias (−1): no study at low risk; two randomized trials at high risk [11,13] and one non-randomized study at serious risk [28]. Inconsistency (−1): I^2 = 88%. Imprecision (−1): interval includes no effect.

CI, confidence interval; SMD, standardized mean difference. Certainty of evidence was assessed using the GRADE approach. Bodies of evidence from randomized trials began at high certainty and those including non-randomized studies at low certainty, and were rated down for risk of bias, inconsistency, indirectness, imprecision, and publication bias. ⨁⨁⨁⨁ high; ⨁⨁⨁○ moderate; ⨁⨁○○ low; ⨁○○○ very low. No outcome was downgraded for indirectness, as all included populations, interventions, and outcomes directly addressed the review question.
